# Extracellular vesicles from regenerative human cardiac cells act as potent immune modulators by priming monocytes

**DOI:** 10.1186/s12951-019-0504-0

**Published:** 2019-05-27

**Authors:** Christien M. Beez, Marion Haag, Oliver Klein, Sophie Van Linthout, Michael Sittinger, Martina Seifert

**Affiliations:** 1 Charité-Universitätsmedizin Berlin, BCRT-Berlin, Institute Of Health Center for Regenerative Therapies, 10178 Berlin, Germany; 2 Institute of Medical Immunology, Charité-Universitätsmedizin Berlin, Corporate Member of Freie Universität Berlin, Humboldt-Universität zu Berlin, and Berlin Institute of Health, Campus Virchow Klinikum (CVK), Foehrer Str. 15, 13353 Berlin, Germany; 3 Tissue Engineering Laboratory, Charité-Universitätsmedizin Berlin, Corporate Member of Freie Universität Berlin, Humboldt-Universität zu Berlin, and Berlin Institute of Health, Berlin, Germany; 4 Department of Internal Medicine and Cardiology, Campus Virchow Klinikum, Charité-Universitätsmedizin Berlin, Corporate Member of Freie Universität Berlin, Humboldt-Universität zu Berlin, and Berlin Institute of Health, Berlin, Germany; 5 DZHK (German Center for Cardiovascular Research), Partner Site, Berlin, Germany; 6 Core Unit Tissue Typing, Charité-Universitätsmedizin Berlin, Corporate Member of Freie Universität Berlin, Humboldt-Universität zu Berlin, and Berlin Institute of Health, 13353 Berlin, Germany

**Keywords:** Extracellular vesicles, Exosomes, Cardiac cells, Immunomodulation, CD14^+^ myeloid suppressive cells, Monocytes

## Abstract

**Background:**

Nano-sized vesicles, so called extracellular vesicles (EVs), from regenerative cardiac cells represent a promising new therapeutic approach to treat cardiovascular diseases. However, it is not yet sufficiently understood how cardiac-derived EVs facilitate their protective effects. Therefore, we investigated the immune modulating capabilities of EVs from human cardiac-derived adherent proliferating (CardAP) cells, which are a unique cell type with proven cardioprotective features.

**Results:**

Differential centrifugation was used to isolate EVs from conditioned medium of unstimulated or cytokine-stimulated (IFNγ, TNFα, IL-1β) CardAP cells. The derived EVs exhibited typical EV-enriched proteins, such as tetraspanins, and diameters mostly of exosomes (< 100 nm). The cytokine stimulation caused CardAP cells to release smaller EVs with a lower integrin ß1 surface expression, while the concentration between both CardAP-EV variants was unaffected. An exposure of either CardAP-EV variant to unstimulated human peripheral blood mononuclear cells (PBMCs) did not induce any T cell proliferation, which indicates a general low immunogenicity. In order to evaluate immune modulating properties, PBMC cultures were stimulated with either Phytohemagglutin or anti-CD3. The treatment of those PBMC cultures with either CardAP-EV variant led to a significant reduction of T cell proliferation, pro-inflammatory cytokine release (IFNγ, TNFα) and increased levels of active TGFβ. Further investigations identified CD14^+^ cells as major recipient cell subset of CardAP–EVs. This interaction caused a significant lower surface expression of HLA-DR, CD86, and increased expression levels of CD206 and PD-L1. Additionally, EV-primed CD14^+^ cells released significantly more IL-1RA. Notably, CardAP-EVs failed to modulate anti-CD3 triggered T cell proliferation and pro-inflammatory cytokine release in monocultures of purified CD3^+^ T cells. Subsequently, the immunosuppressive feature of CardAP-EVs was restored when anti-CD3 stimulated purified CD3^+^ T cells were co-cultured with EV-primed CD14^+^ cells. Beside attenuated T cell proliferation, those cultures also exhibited a significant increased proportion of regulatory T cells.

**Conclusions:**

CardAP-EVs have useful characteristics that could contribute to enhanced regeneration in damaged cardiac tissue by limiting unwanted inflammatory processes. It was shown that the priming of CD14^+^ immune cells by CardAP-EVs towards a regulatory type is an essential step to attenuate significantly T cell proliferation and pro-inflammatory cytokine release in vitro.

**Electronic supplementary material:**

The online version of this article (10.1186/s12951-019-0504-0) contains supplementary material, which is available to authorized users.

## Background

Cardiovascular diseases (CVD) are the leading cause of death worldwide [1] and deteriorate considerably the quality of many patient’s life. To enhance prognosis and wellbeing of patients, a variety of cells were evaluated for their therapeutic potential in experimental and clinical studies, like cardiosphere-derived cells, cardiac progenitor cells, and mesenchymal stromal (MSC) cells from bone marrow, adipose tissue and other sources [2–8]. Another unique cardiac cell type are the human cardiac-derived adherent proliferating (CardAP) cells, which are generated by outgrowth cultures of endomyocardial biopsies [9]. In a set of in vitro assays, it could be shown that CardAP cells are pro-angiogenic, anti-apoptotic and capable to modulate immune responses [10–12]. Furthermore, their administration improved significantly heart functions and impaired immune responses in vivo [12, 13]. Beside these beneficial effects, CardAP cells also have additional advantages in comparison to other regenerative cell types. Firstly, the heart already primes CardAP cells. Histone modifications of cardiac-specific genes were shown to be different between murine cardiac-derived cells and murine bone marrow MSCs, which was correlated to an higher potential for cardiomyogenesis of cardiac than non-cardiac cells [14]. Secondly, CardAP cells are predominantly negative for the membrane glycoprotein CD90 [9]. It was shown in a retrospective analysis of a clinical trial that the therapeutic benefit of applied cardiosphere-derived cells was negatively correlated to the expression of CD90 [15]. Thus, CardAP cells appear very useful for a therapeutic approach to treat CVD patients.

Initially, the observed beneficial effects of the different regenerative cell types were proposed to be facilitated by cell differentiation and integration into the damaged heart [16–18]. However, this mechanism could not be verified as studies failed to show a sufficient retention of therapeutically applied cells in the myocardium [16, 19]. Instead, improved heart functions were induced in the absence of cells, when just their conditioned medium was applied in animal myocardial infarction models [20–22]. Subsequently, investigations focused on the released factors from regenerative cells.

Next to growth factors, cytokines and other proteins, nano-sized extracellular vesicles (EVs) were identified. These lipid bilayer structures can be discriminated in three subsets by their diameter and biogenesis. Apoptotic bodies (> 1 µm) and microvesicles (1– .1 µm) are released by buddying from the cell membrane, whereas exosomes (< .1 µm) originate from intracellularly located multivesicular bodies that fuse to the plasma membrane before they are released into the extracellular space. All three EV types act as potent intercellular communicators by delivering proteins, lipids, RNA and other molecules to a recipient cell [23–29]. Indeed, several studies showed that EVs derived from different cardiac cell types, like cardiomyocytes, endothelial cells, cardiac fibroblasts or cardiac progenitor cells, are able to mediate protective and regenerative effects in injured heart tissue [26, 28–32]. These studies mainly focused on the potential of cardiac-derived EVs to improve heart function by anti-fibrotic, anti-apoptotic, pro-angiogenic and proliferation-inducing effects. All these observed beneficial effects contribute to the opinion that EVs are a very promising therapeutic tool and could eventually replace cellular therapies by application of allogeneic or autologous EVs to CVD patients. For an allogeneic approach, however, it would be necessary to evaluate their immunogenicity to guarantee their future safe usage in humans. Unfortunately, the interaction between the immune system and cardiac-derived EVs has rarely been studied. Furthermore, the ability to limit pro-inflammatory immune responses by immunomodulation can dramatically contribute to the therapeutic success, because lasting inflammation in the heart tissue has opposing effects on the regenerative process [33].

Data already available for EVs from regenerative non-cardiac cells illustrate that these EVs are actually capable of combining low immunogenicity with immune modulating features. This includes in vitro observations like the suppression of induced T cell proliferation and cytokine release [34–37] as well as the induction of regulatory T cells [37, 38] by applied EVs from human or murine MSCs isolated from the bone marrow or umbilical cord. In a murine graft-versus-host-disease model, it could be demonstrated that human embryonic stem cell-derived MSC-EVs alleviated disease symptoms, which was attributed to an induced generation of regulatory CD4^+^ T cells by allogeneic antigen presenting cells [39].

Furthermore, it was shown that human bone marrow-derived MSC-EVs were able to influence antigen presenting cells, like monocyte-derived macrophages in a lung injury model [40] or dendritic cells in vitro [41]. Additionally, it could be demonstrated that human adipose tissue-derived MSC-EVs preferably bind to human monocytes in vitro and induced the apoptosis of the pro–inflammatory CD14^+^CD16^+^ subset, which led to their decreased proportion in the myeloid compartment [42].

To gain a better understanding regarding the effects and mechanisms of EVs from regenerative cardiac cells, we evaluated their impact on clinically relevant inflammatory immune responses by a set of in vitro assays. Therefore, we isolated EVs from the conditioned medium of cardioprotective CardAP cells cultured in the presence or absence of pro-inflammatory cytokines. This cytokine stimulation was applied to enhance anticipated immune modulating effects, since non-cardiac EVs were shown to profit from such a stimulation [34, 43]. Also the immune modulating effects of CardAP cells were only observed in pro-inflammatory milieus [11–13].

Our in vitro results indicate that unstimulated as well as cytokine stimulated CardAP-EVs have a generally low immunogenicity and the therapeutic potential for enhancing cardiac tissue regeneration by limiting undesirable pro-inflammatory immune cell activation.

## Results

### CardAP cells release altered EVs upon cytokine stimulation

**Fig. 1:**
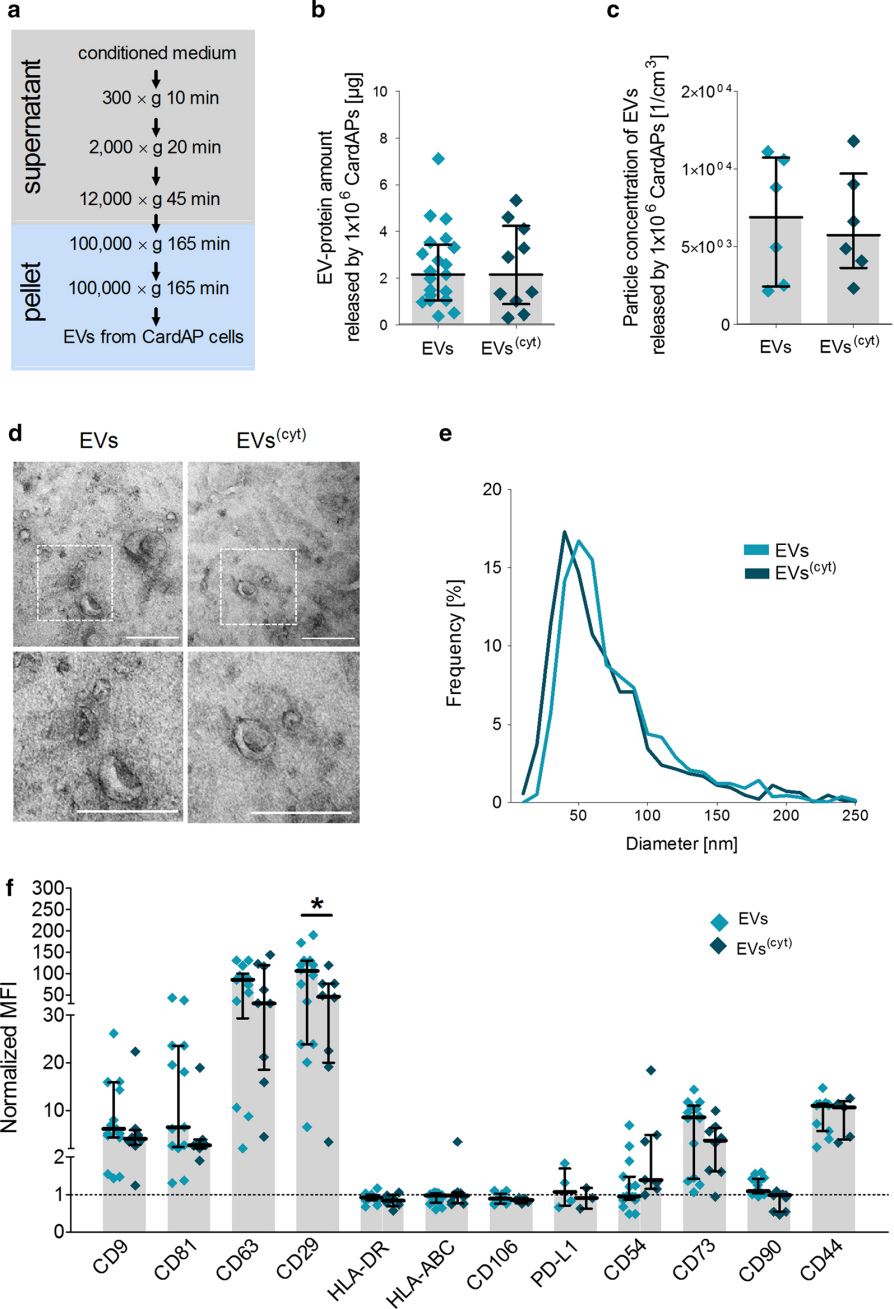
Inflammatory cues change the phenotype of CardAP-EVs. **a** The differential ultracentrifugation protocol is shown to isolate EVs from the conditioned medium under unstimulated (EVs) or cytokine stimulated conditions (EVs^(cyt)^). **b** EV protein amount released from 1 × 10^6^ CardAP cells is presented as median with interquartile range (n = 10–21; six different CardAP donors). **c** Particle concentration of EVs released by 1 × 10^6^ CardAP cells is presented as median with interquartile range (n = 6; three different CardAP donors). **d** Representative transmission electron microscopy (TEM) images (upper row) with an enlarged region (white square) of interest (lower row) are displayed for both EV variants; scale bars represent 200 nm. **e** The diameter distribution as observed by TEM is shown for both EV variants of one CardAP donor. **f** Flow cytometric analyses are presented as median with interquartile range of normalized geometrical mean fluorescence intensities (normalized MFI calculated as ratio of stained to unstained) for tetraspanins (CD9, CD81, CD63), immunological relevant markers (CD54, PD-L1, CD106, HLA-ABC, HLA-DR) and mesenchymal markers (CD29, CD73, CD44, CD90) (n = 5–16; at least three different CardAP donors). Mann–Whitney U-test; ***p < .001, **p < .01, *p < .05

CardAP-EVs were isolated by a stepwise centrifugation of the conditioned media (Fig. 1a), which was derived under either an unstimulated or a pro-inflammatory cytokine stimulation condition (INFγ, TNFα, IL-1β). Indeed, CardAP cells express receptors for the three cytokines and the culturing with cytokines for 20 h statistically enhanced the expression of immunologically relevant markers on the surface of CardAP cells, such as vascular cell adhesion protein-1 (CD106), programmed death ligand 1 and 2 (PD-L1/2) and intercellular adhesion molecule-1 (CD54), while their spindle-shaped morphology and typical mesenchymal markers were unaffected (Additional file 1: Figure S1).

Neither the EV protein amount released by 1 × 10^6^ CardAP cells as determined by BCA assay (Fig. 1b) nor the particle concentration of EVs released by 1 × 10^6^ CardAP cells as determined by NTA (Fig. 1c) was influenced by the cytokine stimulation. Both CardAP-EV variants exhibited also typical sphere-like shapes in sizes ranging from 21 to 295 nm as observed by TEM (Fig. 1d). Quantitative analysis of the determined diameters demonstrated an asymmetrical distribution (Fig. 1e), while most vesicles from both variants (at least 82%) were smaller than 100 nm. Unstimulated EVs (median = 73.80 nm) appeared to be larger in their median diameter in comparison to cytokine stimulated EVs (median = 67.14 nm), (Fig. 1e). This difference between the median diameters of both CardAP-EV variants was further verified by NTA (Additional file 1: Figure S2). Typical EV markers, like Ecto-5′-nucleotidase (CD73), integrin ß1 (CD29) and the tetraspanins CD9, CD81 and CD63, were detected on isolated CardAP-EVs by flow cytometry (Fig. 1f). Unstimulated CardAP-EVs showed a statistically enriched level of CD29 in comparison to cytokine stimulated EVs. Immunological markers, such as HLA-ABC, HLA-DR, CD106 and PD-L1, were not or at very low levels detectable (Fig. 1f).

**Fig. 2:**
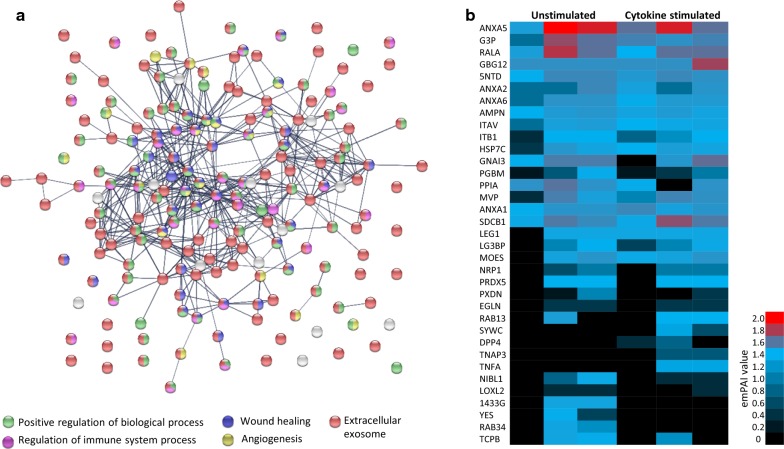
The inflammatory cue changes slightly the proteome of CardAP-EVs. Peptides were derived from unstimulated EVs (unstimulated) and cytokine stimulated EVs (cytokine stimulated) by an overnight digestion with trypsin. By liquid chromatography/electron spray ionization mass spectrometry (LC/ES MS) obtained mass spectra were evaluated by MASCOT software searching for protein matches in the SwissProt 51.9 database. **a** The interaction of proteins was visualized with the help of String database. Protein interactions were shown as connecting lines and were categorized as known interactions (grey connecting lines). The detected proteins are shown as nodes, which appear coloured due to their biological process or localisation (extracellular exosome = red, positive regulation of cellular process = green, angiogenesis = yellow, wound healing = blue, regulation of immune system process = magenta). **b** Shown is a heat map of the exponentially modified protein abundance index (emPAI) for 35 selected proteins from in total 186 detected proteins and for both EV variants from three CardAP donors. Not detected proteins corresponds to an emPAI value of 0

CD73 and CD29 were also detected on both CardAP-EV variants from three different CardAP donors by a liquid chromatograph/electron spray ionisation mass spectrometry (LC/ESI–MS) approach. In total 186 proteins were identified and their interaction as well as functional enrichment in biological processes or cellular compartment was analysed by using String Network Database (Fig. 2a, Additional file 1: Table S1). Generally, most proteins (156/186) could be assigned to the extracellular exosome compartment, like heat shock proteins, integrins or enzymes (e.g. GAPDH). Additionally, CardAP-EVs exhibited proteins that are involved in biological processes, such as angiogenesis (e.g. heparan sulfate proteoglycan 2, neuropillin), wound healing (endoglin, annexin5) or immune system processes (annexin 1, galectin 1). Comparative analysis of the determined exponentially modified protein abundance index (emPAI) between all three CardAP donors reveals that several proteins were identified in all samples, like CD73, while others seem to be donor-specific (Fig. 2b). Furthermore, proteins were exclusively identified for cytokine stimulated EVs, such as TNFα inducible protein and dipeptidyl peptidase 4, or on unstimulated EVs, like Ras-related protein Rab-34 or tyrosine-protein kinase Yes (Fig. 2b).

### CardAP-EVs attenuate pro-inflammatory immune responses in PBMC cultures

**Fig. 3:**
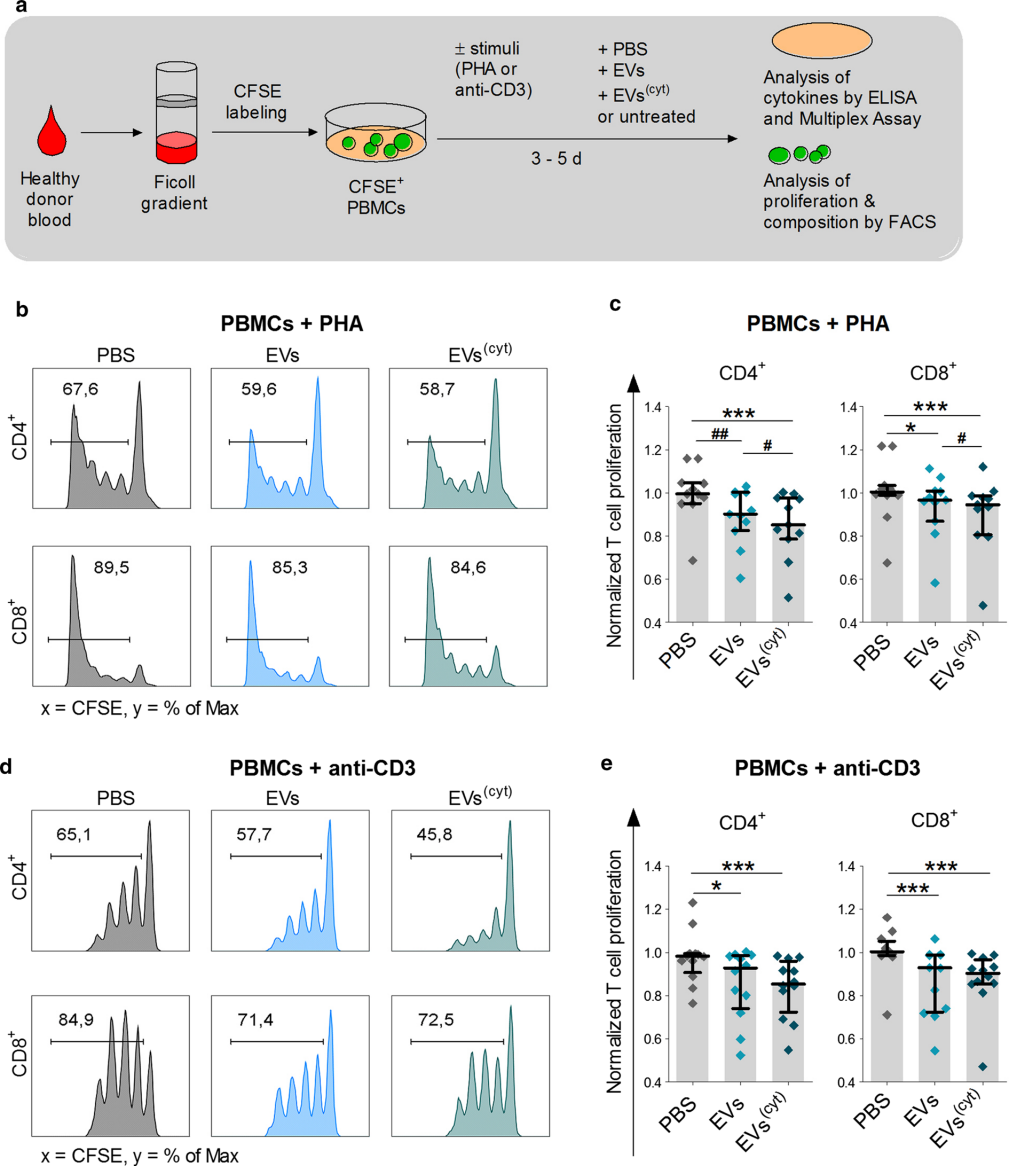
CardAP-EVs diminish PHA and anti-CD3 induced T cell proliferation in PBMC cultures. 3x10^5^ CFSE-labelled PBMCs were stimulated with PHA or anti-CD3, treated with either unstimulated (EVs) or cytokine stimulated (EVs^(cyt)^) EVs, PBS in equal volume of the EVs (PBS) or left untreated and analysed after 3–5 days by flow cytometry. T cell proliferation frequencies were normalized to the untreated control. **a** The general immune assay design is shown. **b**, **d** Representative flow cytometric plots display the frequencies of proliferated CD4^+^ and CD8^+^ T cells in PHA stimulated PBMCs (**b**) or in in anti-CD3 stimulated PBMCs (**d**). The normalized proliferation of CD4^+^ (left) and CD8^+^ (right) T cells in PHA stimulated PBMCs (**c**) or in anti-CD3 stimulated PBMCs (**e**) is presented for the treatment with either CardAP-EV variant and PBS as median with interquartile range (PHA n = 11; four different CardAP donors; five different PBMC donors) (anti-CD3 n = 9; four different CardAP donors; five different PBMC donors). Friedman Test with Dunn’s multiple comparison test: ***p < .001, **p < .01, *p < .05 or Wilcoxon matched-pairs signed rank test; ^###^p < .001, ^##^p < .01, ^#^p < .05

In vitro immune assays are an important initial tool to evaluate future therapies for their immunogenicity and immune modulating capabilities [44, 45]. For that purpose, we isolated human PBMCs from healthy donors and treated them with one of either EV variant, PBS or they were left untreated in the absence or presence of different T cell stimuli (Fig. 3a).

**Fig. 4:**
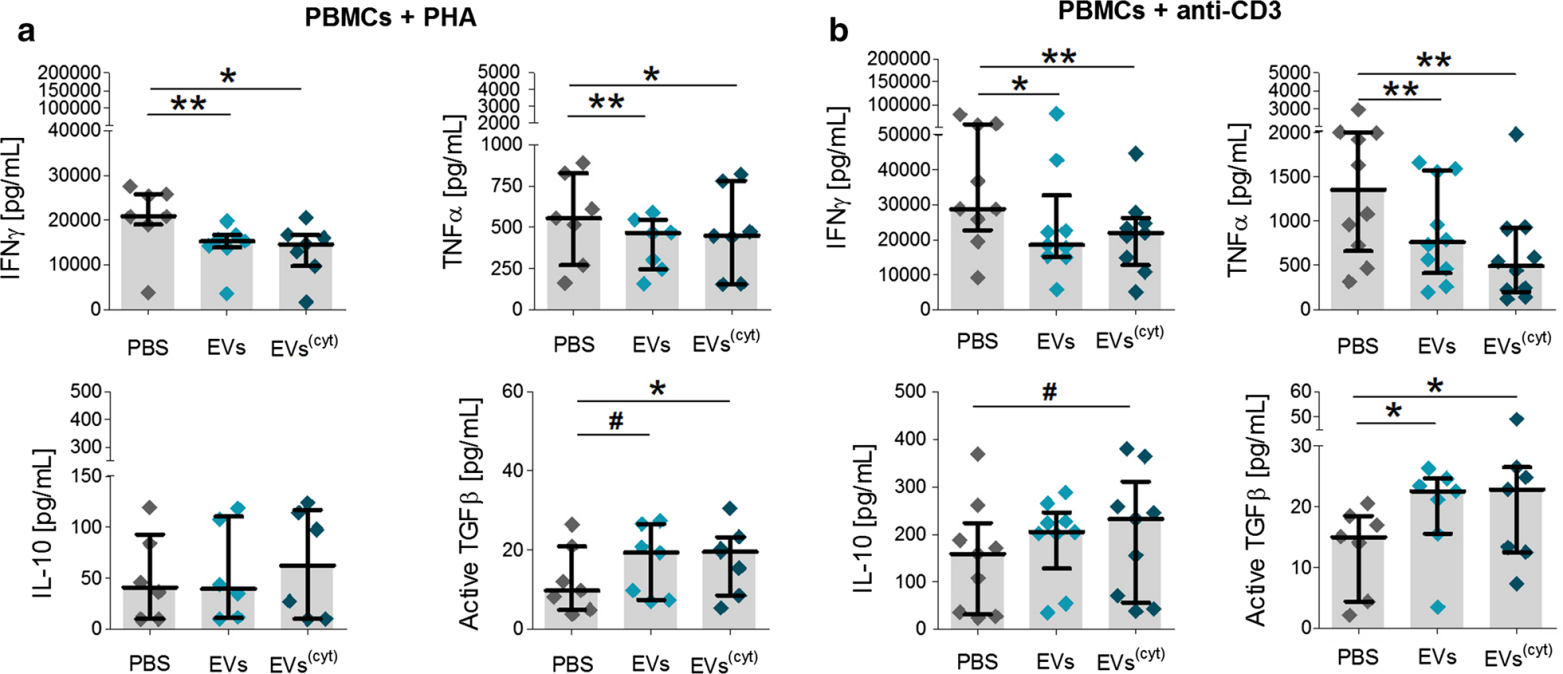
CardAP-EVs attenuate the PHA and anti-CD3 induced pro-inflammatory cytokine release in PBMC cultures. 3x10^5^ CFSE-labelled PBMCs were stimulated with PHA or anti-CD3 antibody and treated with either unstimulated (EVs) or cytokine stimulated (EVs^(cyt)^) EVs, PBS in equal volume of the EVs (PBS) or left untreated and analysed after 3–5 days. The cytokines of the supernatants were analysed by ELISA (IFNγ, active TGFβ) or Multiplex (IL-10, TNFα). Concentrations for all tested cytokines are presented for PHA stimulated PBMC cultures (**a**) or anti-CD3 stimulated PBMC cultures (**b**) as median with interquartile range (PHA n = 6–7, five different CardAP donors, five different PBMC donors) (anti-CD3 n = 8; four different CardAP donors, four different PBMC donors). Friedman Test with Dunn´s multiple comparison test: ***p < .001, **p < .01, *p < .05 or Wilcoxon matched-pairs signed rank test; ^###^p < .001, ^##^p < .01, ^#^p < .05

Both CardAP-EV variants exhibited a generally low immunogenicity, as a 5-day exposure of unstimulated PBMCs to each EV variant did not induce any T cell proliferation (Additional file 1: Figure S3). On the other hand, the treatment with either CardAP-EV variant significantly reduced the CD4^+^ and CD8^+^ T cell proliferation in PBMC cultures stimulated with either Phytohemagglutin (PHA) (Fig. 3b, c) or anti-CD3 (Fig. 3d, e) in comparison to the respective PBS control. Interestingly, the CD4^+^ and CD8^+^ T cell proliferation was even greater diminished in PHA stimulated PBMC cultures treated with cytokine stimulated CardAP-EVs (median .85 CD4^+^; median .94 CD8^+^) than with unstimulated CardAP-EVs (median .90 CD4^+^, median .96 CD8^+^). A similar trend was detcted in anti-CD3 stimulated PBMCs. The treatment with either EV variant also contributed towards a less pro-inflammatory cytokine profile under PHA stimulation (Fig. 4a) or anti-CD3 stimulation (Fig. 4b), as observed by significant lower IFNγ as well as TNFα levels and a higher level of active TGFβ. The IL-17a release in PHA stimulated PBMCs and IL-1ß in anti-CD3 stimulated PBMCs were reduced by trend upon CardAP-EV treatment (Additional file 1: Figure S4). Additionally, IL–10 was increased in PBMC cultures treated with cytokine stimulated CardAP-EVs by trend under PHA stimulation (Fig. 4a) and statistically significant under anti-CD3 stimulation (Fig. 4b) in comparison to the respective PBS controls.

### CardAP-EVs primarily target CD14^+^ cells and induce a regulatory phenotype

**Fig. 5:**
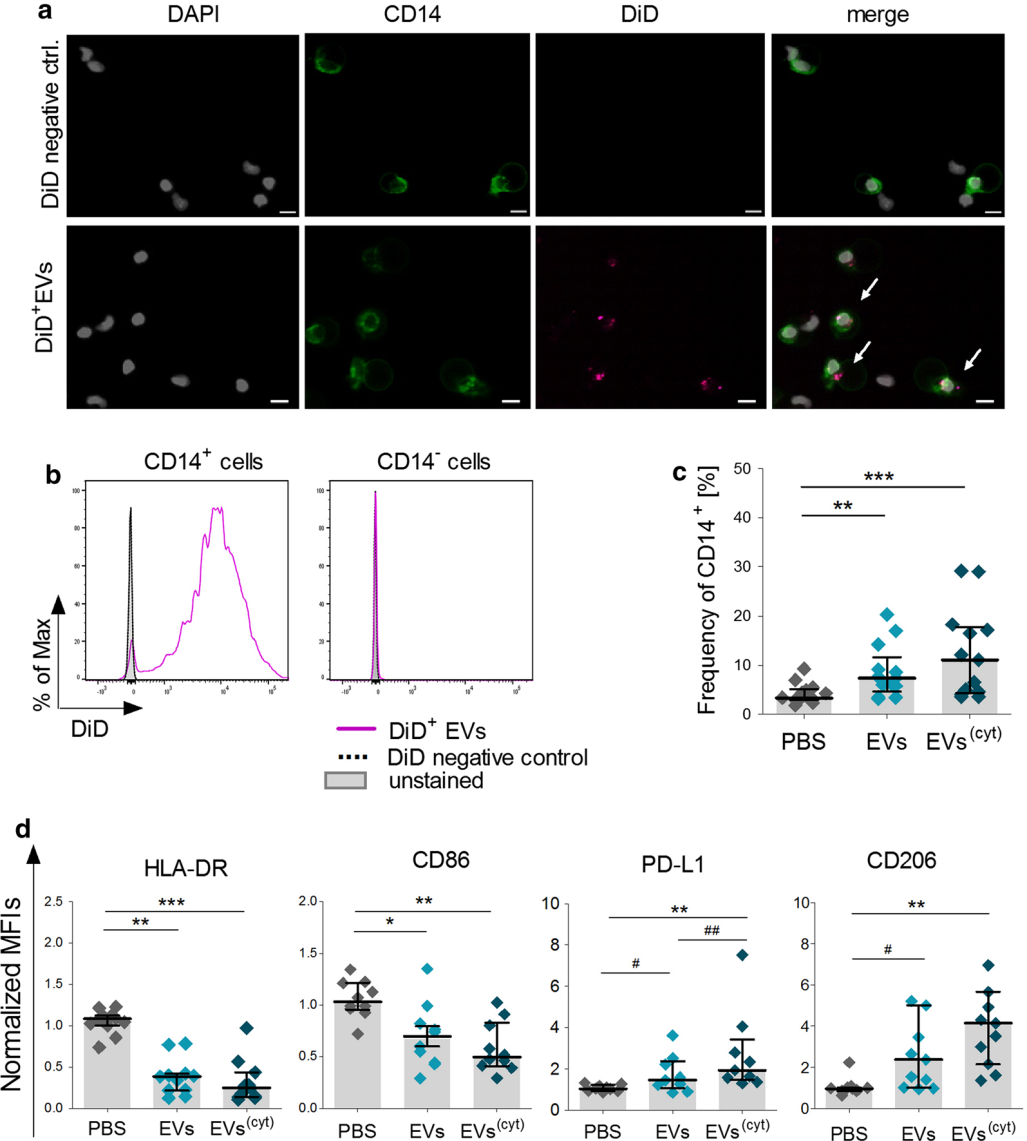
CardAP-EVs prime CD14^+^ monocytes in unstimulated PBMC cultures towards a regulatory CD14^+^ myeloid cell type. 1x10^6^ PBMCs were treated with DiD-labelled CardAP-EVs for 24 h and analysed by microscopy or flow cytometry. **a** Representative images are illustrating co-localization (white arrows) of DiD^+^EVs (magenta) with CD14^+^ PBMCs (green) in total PBMCs (pseudo coloured white for DAPI) (n = 2; three different CardAP donors, two different PBMC donors). Scale bars represent 10 µm. **b** Representative histograms of the flow cytometric analysis are shown for CD14^+^ and CD14^−^ immune cells (n = 2; three CardAP donors; two different PBMC donors). For the phenotypical analysis, 1x10^6^ PBMCs were treated with unstimulated (EVs) or cytokine stimulated EVs (EVs^(cyt)^), PBS in equal volume of the EVs (PBS) or left untreated. After 3 days, cells were analysed by flow cytometry. **c** Frequencies of CD14^+^ cells in PBMCs are presented for cultures treated with PBS, EVs or EVs^(cyt)^. **d** Flow cytometric surface expression data are presented as median with interquartile range of normalized geometrical mean fluorescence intensities (normalized MFI calculated as ratio of stained to unstained) for the immunological markers HLA-DR, CD86, PD-L1 and CD206 (n = 11; four CardAP donors, four PBMC donors). Friedman Test with Dunn´s multiple comparison test: ***p < .001, **p < .01, *p < .05 or Wilcoxon matched-pairs signed rank test; ^###^p < .001, ^##^p < .01, ^#^p < .05

To unravel the recipient cell of CardAP-EVs, total PBMC cultures were treated with fluorescently labelled CardAP-EVs (DiD^+^EVs) for 24 h. Flow cytometric analysis clearly illustrated that DiD^+^EVs were primarily interacting with CD14^+^ cells (96.6% DiD^+^ cells) rather than with CD14^−^ immune cells (2.8% DiD^+^ cells) (Fig. 5b), which we could also verify by the co-localization of both signals (Fig. 5a). After 3 days, the frequency of CD14^+^ cells was significantly increased in unstimulated PBMC cultures treated with either CardAP-EV variant (Fig. 5c) respectively to the PBS controls. Furthermore, the phenotype of EV-primed CD14^+^ cells changed dramatically. Both CardAP-EV variants (Fig. 5d) raised significantly the surface expression levels of PD-L1 and significantly lowered the expression levels of HLA-DR and CD86 in comparison to the PBS controls. Also CardAP-EVs significantly enhanced the surface expression levels of the macrophage mannose receptor 1 (CD206). Notably, the expression of PD–L1 was significantly more enhanced by cytokine stimulated CardAP-EVs in comparison to the unstimulated counterpart.

### EV-primed CD14^+^ cells are necessary to suppress immune responses

**Fig. 6:**
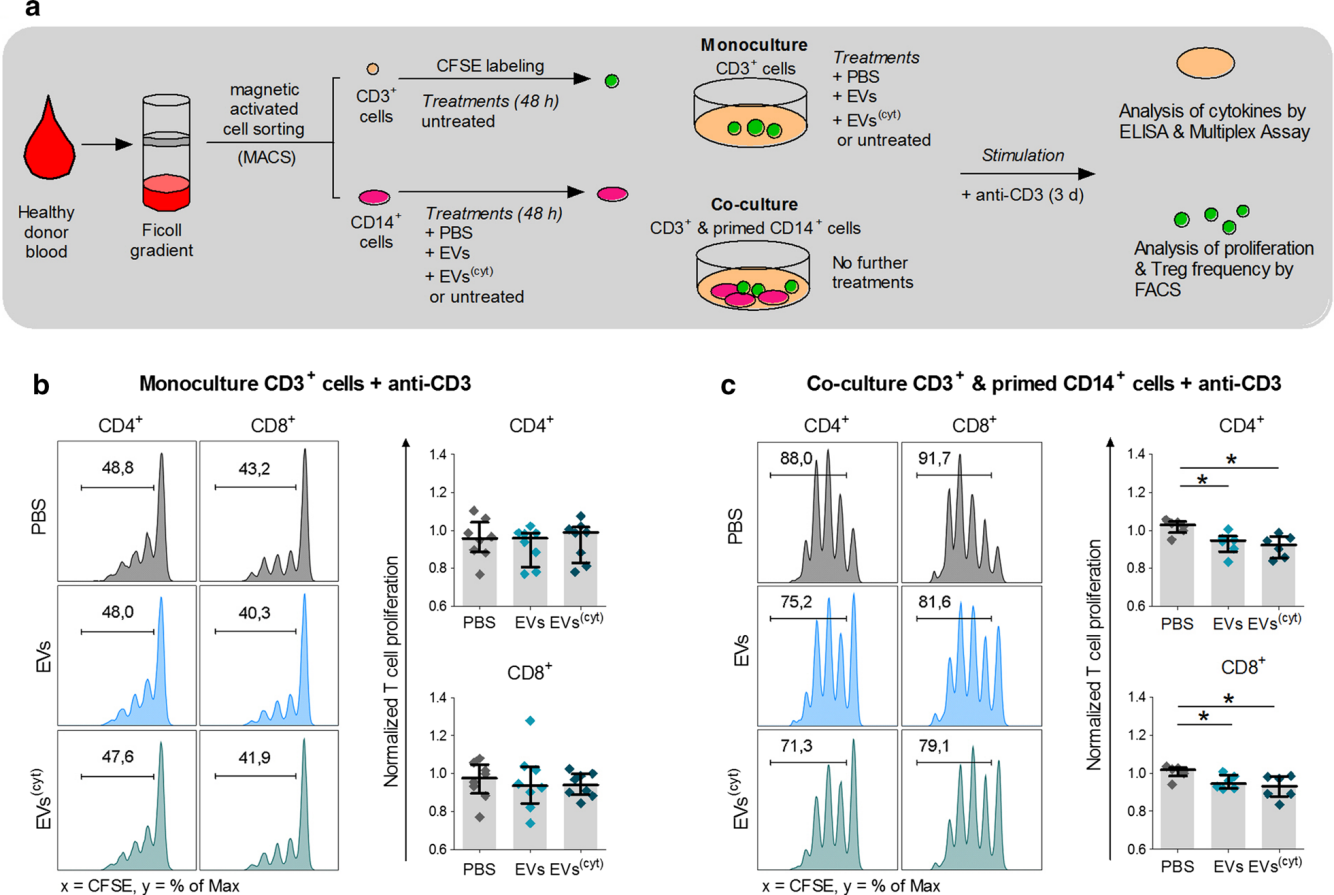
CardAP-EVs diminish anti-CD3 induced T cell proliferation only in the presence of CD14^+^ cells. CD3^+^ and CD14^+^ cells were isolated by MACS and cultured unstimulated for 48 h. Here, CD14^+^ cells were additionally treated with either unstimulated (EVs) or cytokine stimulated (EVs^(cyt)^) EVs, PBS in equal volume of the EVs (PBS) or left untreated. Afterwards, CD14^+^ cells were co-cultured 1:5 with CD3^+^ cells and stimulated with anti-CD3. Additionally, a negative control was incorporated by treating anti-CD3 stimulated CD3^+^ cells with either CardAP-EV variant, PBS or left untreated. After 3 days, the cells were harvested and analysed by flow cytometry. T cell proliferation frequencies were normalized to the untreated control. **a** The general immune assay design is shown. Representative flow cytometric plots display the frequencies of proliferated CD4^+^ and CD8^+^ T cells in anti-CD3 stimulated monocultures of CD3^+^ cells (**b**, left) and anti-CD3 stimulated co-cultures of CD3^+^ cells with primed CD14^+^ cells (**c**, left). The normalized proliferation of CD4^+^ (upper graph) and CD8^+^ (lower graph) T cells in anti-CD3 stimulated monocultures of CD3^+^ cells (**b**, right) or in co-culture with primed CD14^+^ cells (**c**, right) is presented for the treatment with either CardAP-EV variant and PBS as median with interquartile range (monocultures n = 7; four different CardAP donors; five different PBMC donors) (co-cultures n = 5; four different CardAP donors; four different PBMC donors). Friedman Test with Dunn’s multiple comparison test: ***p < .001, **p < .01, *p < .05 or Wilcoxon matched-pairs signed rank test; ^###^p < .001, ^##^p < .01, ^#^p < .05

**Fig. 7:**
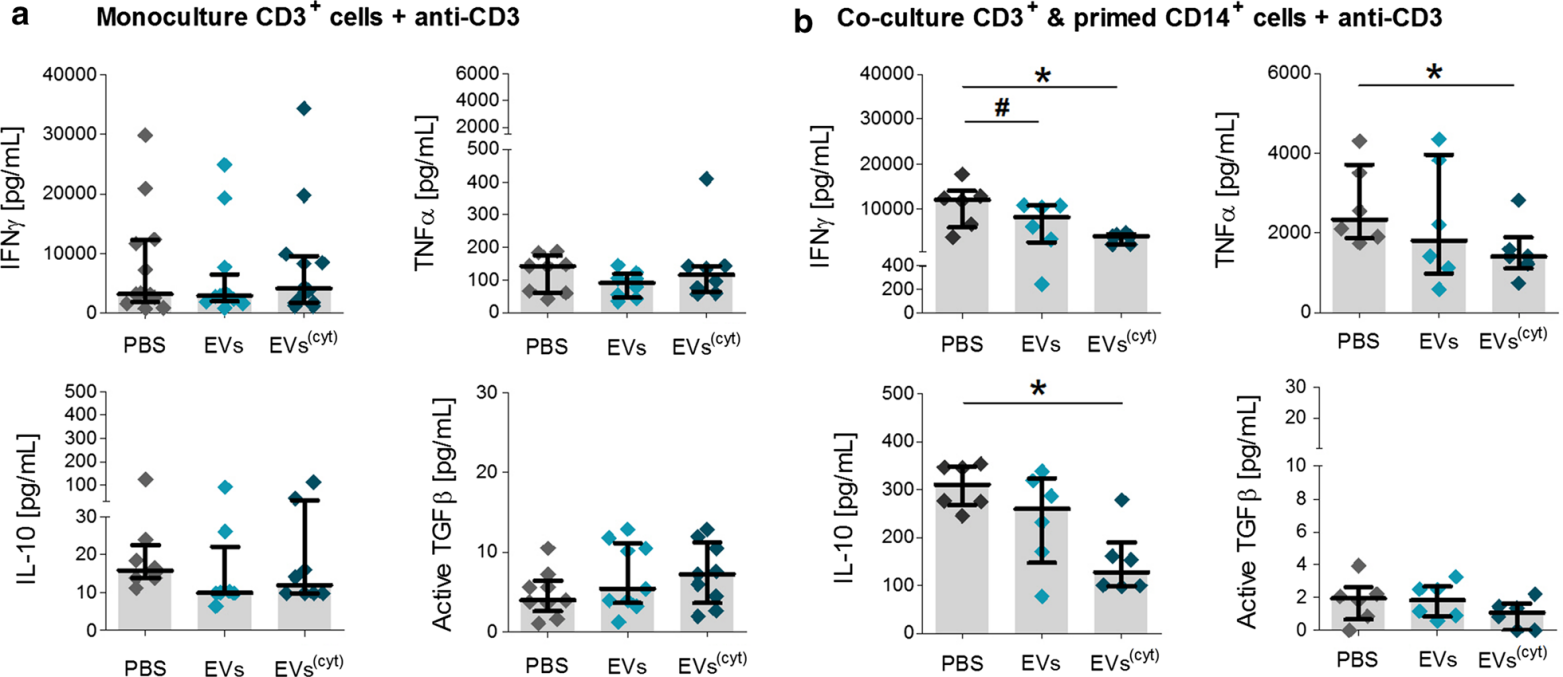
CardAP-EVs attenuate anti-CD3 induced pro-inflammatory cytokine release only in the presence of CD14^+^ cells. CD3^+^ and CD14^+^ cells were isolated by MACS and cultured unstimulated for 48 h. Here, CD14^+^ cells were additionally treated with either unstimulated (EVs) or cytokine stimulated (EVs^(cyt)^) EVs, PBS in equal volume of the EVs (PBS) or left untreated. After 2 days, CD14^+^ cells were co-cultured 1:5 with CD3^+^ cells and stimulated with anti-CD3. As a negative control, anti-CD3 stimulated T cells were treated with either CardAP-EV variant, PBS or left untreated. After 3 days, the supernatants were collected and cytokine concentrations were analysed by ELISA (IFNγ, active TGFβ) or Multiplex (IL-10, TNFα). Concentrations for all tested cytokines are presented for anti-CD3 stimulated monoculture of CD3^+^ cells (**a**) or co-cultures of CD3^+^ cells with CD14^+^ cells (**b**) as median with interquartile range (co-culture n = 6, five different CardAP donors, five different PBMC donors) (monoculture n = 8; four different CardAP donors, four different PBMC donors). Friedman Test with Dunn´s multiple comparison test: ***p < .001, **p < .01, *p < .05 or Wilcoxon matched-pairs signed rank test; ^###^p < .001, ^##^p < .01, ^#^p < .05

**Fig. 8:**
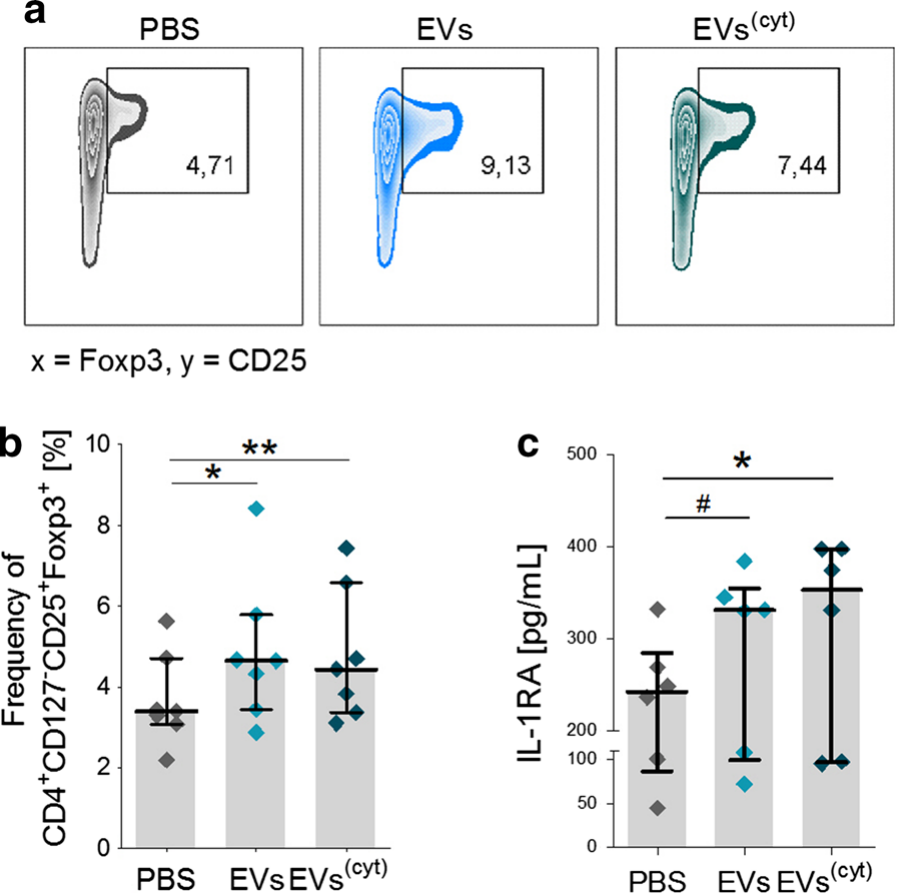
CardAP-EVs increase the frequency of regulatory T cells in anti-CD3 induced co-cultures of CD3^+^ cells with primed CD14^+^ cells. CD3^+^ and CD14^+^ cells were isolated by MACS and cultured unstimulated for 48 h. Here, CD14^+^ cells were additionally treated with either unstimulated (EVs) or cytokine stimulated (EVs^(cyt)^) EVs, PBS in equal volume of the EVs (PBS) or left untreated. Afterwards, CD14^+^ cells were co-cultured 1:5 with CD3^+^ cells and stimulated with anti-CD3. After 3 days, the cells were harvested and analysed by flow cytometry. **a** Representative flow cytometric plots (Foxp3 vs CD25) display the frequencies of regulatory T cells (CD4^+^CD197^−^CD25^+^Foxp3^+^) in anti-CD3 stimulated co-cultures of CD3^+^ cells and CD14^+^ cells primed with PBS (left), EVs (middle) and EVs^(cyt)^ (right). **b** Quantitative analysis of regulatory T cell frequency is shown as median with interquartile range for the treatment with either CardAP-EV variant and PBS (n = 6; four different CardAP donors; four different PBMC donors). **c** Concentration of released IL-1RA by CD14^+^ cells after 2-days treatment with either CardAP EV variant or PBS is shown as median with interquartile range (n = 6, four different CardAP donors, four different PBMC donors). Friedman Test with Dunn´s multiple comparison test: ***p < .001, **p < .01, *p < .05 or Wilcoxon matched-pairs signed rank test; ^###^p < .001, ^##^p < .01, ^#^p < .05

The question arouse whether EV-primed CD14^+^ cells are playing a role in the previously described immune suppressive effects in PBMC cultures. Therefore, we adjusted the assay by using purified CD3^+^ T cells and CD14^+^ monocytes, generated by MACS sorting, instead of PBMCs. The purified CD3^+^ T cells were stimulated with anti-CD3 for 3 days and either cultured with EV-primed CD14^+^ cells or treated with CardAP-EV corresponding controls (Fig. 6a). Anti-CD3 induced CD4^+^ or CD8^+^ T cell proliferation was not affected in isolated CD3^+^ T cell cultures treated with either CardAP-EV variant in respect to the PBS-control (Fig. 6b). In contrast, combination of purified CD3^+^ T cells with EV-primed CD14^+^ cells resulted in a significant reduction of CD4^+^ as well as CD8^+^ T cell proliferation (Fig. 6c). Likewise, no changes in the amount of released IFNγ or TNFα were detected in the purified CD3^+^ T cells culture setting (Fig. 7a), while significant lower levels of pro-inflammatory IFNγ were detected in co-cultures of CD3^+^ T cells with EV-primed CD14^+^ cells (Fig. 7b). TNFα was significantly reduced by CD14^+^ cells primed by cytokine stimulated CardAP-EVs and a similar effect is visible by trend for CD14^+^ cells treated with unstimulated CardAP EVs prior to the co-culture with T cells (Fig. 7b). Interestingly, the IL-10 level was found to be significantly reduced in co-cultures with CD14^+^ cells that were primed by cytokine stimulated CardAP-EVs, whereas active TGFß was detectable on very low levels (Fig. 7b). Nevertheless, the frequency of regulatory T cells (CD4^+^CD127^−^CD25^+^Foxp3^+^) was significantly enhanced in those co-cultures with EV-primed CD14^+^ cells in comparison to the control setting of PBS-treated CD14^+^ cells (Fig. 8a, b). Analysis of supernatants from 48 h primed CD14^+^ cells showed that interleukin 1 receptor antagonist (IL-1RA) has been significantly enhanced (Fig. 8c), while IL-1β, IFNγ and IL-10 were not detectable in those supernatants (data not shown).

## Discussion

Cardiac-derived cells, like CardAP cells, demonstrated a great potential to treat CVD by pro-angiogenic, anti-fibrotic, anti-apoptotic and immune modulating features [9–13, 46–48]. Over the last years, evidence accumulated that paracrine modulators, especially extracellular vesicles, are key players of those regenerative effects [28, 49, 50]. There is, however, just very few information available whether cardiac-derived EVs are able to liberate regenerative processes by limiting chronic inflammation in the cardiac tissue. Therefore, we investigated the capacity of unstimulated or pro-inflammatory stimulated CardAP-EVs to modulate crucial immune responses in vitro.

We could show for the first time to our knowledge that cardiac–derived EVs are potent modulators of CD14^+^ monocytes by shifting their surface marker profile towards a rather immune regulatory one and that solely the presence of EV-primed CD14^+^ cells is capable to attenuate adaptive T cell immune responses.

Both CardAP-EV variants demonstrated typical EV characteristics and a low immunogenic phenotype. The protein amount as well as the particle concentration of EVs released by 1 × 10^6^ CardAP cells was comparable between both conditions. But their size and marker expression differed significantly. The majority of CardAP-EVs exhibited dominantly diameter measures of exosomes, while cytokine stimulated EVs showed a smaller median diameter. This observation has not been described yet and might be influenced by an altered composition of exosomes. Exosomes have been described to be a heterogeneous EV population, including their size and transported proteins [23, 51]. It is further corroborated by a significant lower CD29 detection on cytokine stimulated EVs as observed by flow cytometry. Furthermore, other proteins were exclusively detected under either cytokine stimulated condition (e.g. TNFα induced protein 3) or unstimulated condition (e.g. Ras-related protein Rab-34) by LC/ESI-MS. This observation has to be validated by analysing a greater number of CardAP donors. The majority of identified proteins (156/186) could be assigned to the extracellular exosome compartment, which also included surface markers already detected by flow cytometry. These typical EV-enriched proteins, such as CD73, were detectable in comparable expression levels on both CardAP-EV variants. A low immunogenicity would be ensured by a very low HLA-ABC expression and the complete absence of HLA–DR, which both were not detectable by LC/EMI-MS. Indeed, this could be verified by an absence of any T cell proliferation response against the applied CardAP-EVs in PBMC cultures. This is in accordance with recent studies, where any T cell proliferation was induced by human embryonic stem cell-derived MSC-EVs in mouse spleenocyte cultures [52] or by EVs from amniotic fluid stem cells in human PBMC cultures [53]. Importantly, we found that EVs interacted pre-dominantly with CD14^+^ cells in those PBMC cultures. So far, evidence is missing whether CardAP-EVs have been internalized. But we would argue that EVs are rather taken up by CD14^+^ cells due to their enhanced phagocytosis capability. Likewise, others described a preferred interaction of CD14^+^ cells with vesicles released by labelled human MSCs derived from the bone marrow in a trans-well culture system [43].

We found that CardAP-EVs treatment changed CD14^+^ cells towards a regulatory phenotype by expressing lower levels of HLA-DR, CD86 and enhanced levels of PD-L1 and CD206. Studies from other groups show similar changes of these cell´s phenotype upon phagocytosis of human umbilical cord MSCs [54] or treatment with EVs from glioma stem cells [55] or human adipose tissue-derived MSCs [42, 56]. Dam et al. for example, observed the down-regulation of HLA-DR and CD86 on isolated monocytes after co-culturing with human CPCs. Interestingly, this effect was facilitated independently of any IFNγ pre-treatment of CPCs [48], which is in accordance to our results. Also Lo Sicco et al. demonstrated a similar phenotype, including significantly increased CD206 and reduced CD86 levels on the surface of isolated macrophages after exposure to human adipose tissue-derived MSC-EVs. Moreover, their application in a cardio-toxin induced injury mouse model enhanced regeneration of the skeletal muscle, which was attributed to the increased detection of M2-type macrophage markers [56]. While de Witte et al. inhibited the adherence of monocytes, we assume that in our study CD14^+^ blood monocytes had already differentiated into macrophages as visible by their treatment-independent adherence [54]. The observed changes of surface molecules on CD14^+^ cells primed by CardAP-EVs would support a partial shift towards the anti-inflammatory M2-type macrophage. It would be further fostered by the increased release of IL-1RA of purified CD14^+^ cells treated with either CardAP-EV variant, while the pro-inflammatory cytokines IL-1ß and IFNγ were not detectable. IL-1RA antagonises IL-1β signalling and thereby suppresses immune responses, which is a known mechanism used by M2-type macrophages [57] or even by murine bone marrow-derived MSCs to induce M2 polarization [58]. In general, the polarization of M2-type macrophages would be beneficial for a potential CardAP-EV product, since the induced macrophages release anti-inflammatory cytokines, chemokines and growth factors [59], which may enhance myocardial repair [60, 61]. The detected increase of the M2-type marker CD206, however, might imply either beneficial effects, like an inflammation-resolving function [62], or detrimental effects by inducing fibrosis [63].

So far, the mechanisms are not yet clear how the EV treatment leads to the detected alterations on CD14^+^ cells, like the down-regulation of HLA-DR. It can be speculated that tetraspanins are involved, since they are well-known players in antigen-presentation and internalization of HLA-DR [64]. But also the delivery of microRNAs by CardAP-EVs might have an effect on these cells, because specific microRNAs are playing a role in the process of macrophage polarization towards M1/M2-type [65]. Several studies confirmed the capacity of EVs from regenerative cells to deliver RNA molecules [29, 66, 67]. It has to be investigated which microRNAs CardAP-EVs do deliver and whether they differ between unstimulated and cytokine stimulated conditions. We assume a reduced antigen-presenting feature of CD14^+^ cells exposed to CardAP-EVs within triggered immune cell cultures, since we observed a down-regulation of HLA-DR and CD86. Indeed, we found that mitogen and antibody induced T cell proliferation was significantly reduced in EV-treated PMBC cultures respectively to controls. EV-mediated effects on T cell proliferation are controversially discussed, as results were obtained for diminished [35, 68] or unaffected [43, 69] T cell proliferation by EVs from glioma stem cells or MSCs from bone marrow or umbilical cord. This might reflect the heterogeneity within study design, such as different EV isolation methods or differences in the conducted immunomodulation assays. In our study, the priming of CD14^+^ myeloid cells by CardAP-EVs was essential, since immune responses were not modulated in their absence. Subsequently, no EV mediated inhibition of T cell proliferation was detectable when solely anti-CD3 stimulated purified CD3^+^ T cells were used in the immunomodulation experiment. In presence of CD14^+^ cells, as seen in the experimental setup with EV-primed CD14^+^ cells combined with purified CD3^+^ T cells, the EV-mediated suppression of T cell proliferation could be restored as we initially observed in whole PMBC cultures. This discrepancy between whole PBMC and purified CD3^+^ T cell cultures was also described for murine immune cells treated with murine bone marrow-derived MSC-EVs [37]. Additionally, we observed that the profile of released cytokines was not altered in cultures of purified T cells, while combined and EV-treated cultures of isolated CD14^+^ with CD3^+^ cells as well as EV-treated total PBMCs displayed reduced pro-inflammatory cytokine levels (IFNγ, TNFα). The reduction of pro-inflammatory cytokines is in line with other publications, showing less release of IFNγ, TNFα, IL-1β or IL-17 even in the absence of inhibiting effects on the T cell proliferation [38, 43, 52, 69]. Furthermore, PBMC cultures displayed enriched levels of active TGFβ after treatment with both EV variants, which emphasizes the reduced inflammatory response towards the applied cues. Other cytokines that confirm this observation are indicated, such as reduced IL-17 or IL-1ß levels while solely cytokine stimulated EVs increased level of IL-10. This anti-inflammatory cytokine was accelerated in previous studies, including treatment with EVs from human bone marrow-derived MSCs or glioma stem cells as well as our own studies from CardAP cells in the in vivo models [12, 38, 55].

The question arises: how do EV-primed CD14^+^ cells contribute to the reduced immune response? We speculate that beside the reduction of antigen presentation, other direct cell–cell-interactions can play a role. The engulfing of PD-1 on the surface of T cells with upregulated PD-L1 on the EV-primed CD14^+^ cells can lead to the inhibition of T cell proliferation and their apoptosis [70]. Also phosphatidylserine might be involved, as its exposure on vesicles released by polymorph nuclear cells was shown to inhibit the differentiation of monocyte-derived dendritic cells and thereby attenuating T cell proliferation [71]. Additionally, paracrine mechanisms can limit immune responses, like the detected increased release of IL-1RA, but also other cytokines, chemokines or EVs from CD14^+^ cells themselves influence the outcome of the immune response [72]. Also, it cannot be ruled out that EV-primed CD14^+^ cells may affect other immune cells than T cells. Likewise, we observed an inferior interaction with the CD14^−^ immune cell subpopulations, therefore we cannot rule out that CardAP-EVs can influence other immune cells directly, as human bone marrow-derived MSC-EVs were shown to influence B cells in purified immune cell cultures [43].

Interestingly, we observed in the MS-analysis of our CardAP-EVs that galectin-1 and several proteins from the annexin family were present. They are known to be modulators of immune responses by binding of galectin-1 to CD69 and imitating a signal cascade which promotes regulatory T cell development [73]. After interaction with macrophages, annexin 1 further promotes a M2-type macrophage polarization [74, 75]. In combined cultures of purified CD14^+^ and CD3^+^ cells an increased frequency of regulatory T cells was detected. The administration of human or murine bone marrow-derived MSC-EVs has been previously shown to enhance regulatory T cells [38, 52, 69], which happened for EVs of embryonic stem cell-derived MSCs in a monocyte-dependent mode of action [52]. The ability of CardAP-EVs to enhance the proportion of regulatory T cells is supported by the presented data, showing increased amounts of active TGFβ in PHA and anti-CD3 stimulated PBMC cultures and increased IL-10 levels in anti-CD3 PBMCs treated with cytokine stimulated CardAP-EVs. Although IL-10 was decreased in co-cultures of CD3^+^ cells with CD14^+^ cells, we found that EV-treated CD14^+^ cells already secreted IL-1RA before adding them to the co-cultures, which is able to induce regulatory T cell development [40]. Supportively, it was shown in vivo that the application of CardAP cells was also increasing the number of regulatory T cells [13].

Based on the discovered immunomodulatory CardAP-EV effects in our study a potential clinical application would be feasible. This might include cardiac diseases like myocardial infarction, ischemic heart diseases, heart failure and atherosclerosis. Moreover, modulation of adverse immune processes could be dampened via the observed shift of CD14^+^ cells into a pro-regenerative phenotype and the enhanced induction of regulatory T cells in selected autoimmune diseases, such as diabetes or arthritis. However, further studies are needed to determine the nature of delivered active molecules, like microRNAs. Furthermore, we exclusively focused on the in-depth characterization of EVs from CardAP cells and in particular the influence of a clinical relevant inflammatory environment. A direct comparison with EVs from other immune modulatory active cells, like cardiac or non-cardiac MSCs, as well as cells with any known immune modulating activity was not performed, but would be very interesting to be considered in future studies to distinguish between broad and specific mechanisms.

## Conclusions

**Fig. 9:**
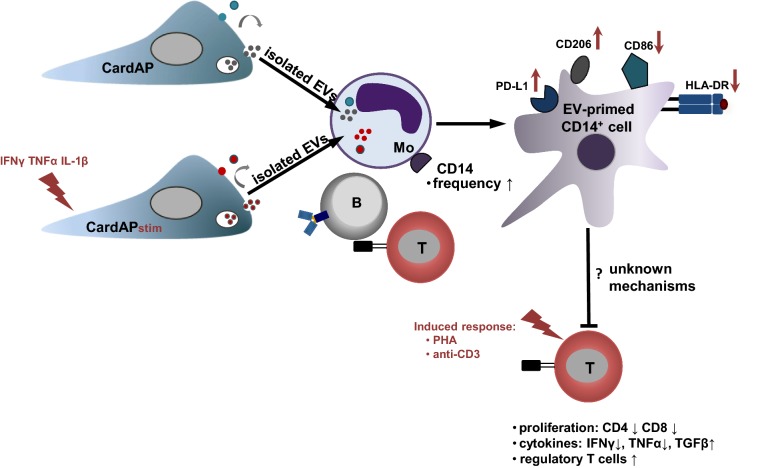
Hypothesized model of the immunomodulatory effects by CardAP-EVs. CardAP cells release EVs under unstimulated or cytokine stimulation (CardAPstim). Isolated EVs interact mainly with the CD14^+^ monocyte subset (Mo), leading to an enhanced CD14^+^ frequency and a changed phenotype marked by a reduced expression of HLA-DR and CD86, but enhanced expression of CD206 and PD-L1. We showed that the development of such a CD14^+^ regulatory myeloid cell subset is mediating the observed attenuated T cell immune responses

We found that CardAP-EVs exhibit a low immunogenicity and a capability to lower significantly pro-inflammatory immune responses in vitro. The present study provides the first evidence that the priming of CD14^+^ cells by cardiac-derived EVs in PBMC cultures is an essential requirement to facilitate their immune modulating features, as detected by attenuated T cell proliferation and pro-inflammatory cytokine release (summarized in Fig. 9). Overall, these findings would support an allogeneic approach of CardAP-EVs to improve cardiac regeneration.

## Methods

An expanded version is available in the additional material online.

### Isolation of CardAP-EVs

Isolation of EVs was performed by differential centrifugation of conditioned medium adopted from the described method by Théry et al. [27]. Shortly, cryopreserved CardAP cells, which were derived by an outgrowth culture from endomyocardial biopsies as previously described [9], were thawed and cultured in IDH medium containing 10% ultracentrifuged human serum (ucIDH), which was prepared by centrifuging IDH medium supplemented with 50% human serum (German Red Cross, Berlin, Germany) for 24 h at 100,000×*g* (L7-55 ultracentrifuge with SW-32 Ti buckets; all from Beckman coulter, Palo Alto, CA, USA).

CardAP cells were grown in ucIDH to a confluence of about 80% and washed twice with phosphate-buffered saline (PBS; Biochrom). Afterwards, cells were either stimulated with 10 ng/mL of human tumor necrosis factor α (TNFα), human interferon-γ (IFNγ) and interleukin 1β (IL-1β; all purchased from Miltenyi Biotec, Bergisch Gladbach, Germany) or unstimulated in serumfree IDH medium. After 20 h under 37 °C and 5% CO_2_, the conditioned medium was collected and the supernatant was stepwise centrifuged at 300×*g* for 10 min, 2000×*g* for 20 min, 12,000×*g* for 45 min and at 100,000×*g* for 165 min (Allegra^®^ X-15R centrifuge and L7-55 ultracentrifuge with SW-32 Ti buckets; all from Beckman coulter). Then, the received EV pellet was washed with .1 µm filtered PBS by repetition of the last ultracentrifugation step. At the end, the received EV pellet was resuspended in 500 µL .1 µm filtered PBS, transferred to low-binding tubes (Sarstedt, Nümbrecht, Germany) and stored at − 80 °C till further usage. CardAP-EVs have been isolated from six different donors in passage three to seven.

### Size and concentration determination of CardAP-EVs

CardAP-EVs were positive-negatively stained [27] and morphologically evaluated by transmission electron microscopy (TEM) at the EM facility of the Charité-Universitätsmedizin Berlin. Briefly, 20 µL of EVs were placed for 20 min on formavor-carbon coated copper EM grids (Electron Microscopy Sciences, Hatfield, PA, USA). Afterwards, following steps were performed: 20 min in 2% paraformaldehyde (Roth, Karlsruhe, Germany), 5 min in 1% glutaralaldehyde (Sigma Aldrich, St. Louis, MO, USA), several washing steps with water and 10 min in freshly prepared 4% uranylacetate 2% methylcellulose (both from Sigma-Aldrich) solution. Samples were analysed by the transmission electron microscope Zeiss Leo 906 (Carl Zeiss Microscopy GmbH, Jena, Germany) run with ImageSP Viewer software version 1.2.7.11 (SYS-PROG, Minsk, Belarus). For each isolation condition, at least 12 individual pictures were accessed for the diameter of EVs by ImageSP Viewer and analysed for their diameter distribution respectively. The concentration and size distribution of EVs was analysed by nanoparticle tracking analysis (NTA). Here samples were measured at ZetaView^®^ (Particle Metrix, Meerbusch, Germany) with the camera level 14 and according manufacture’s manual. The particle concentration of EVs/1 × 10^6^ CardAP cells was calculated. Furthermore, the protein content was determined by Pierce™ BCA protein assay (Thermo Scientific, Rockford, IL, USA) according to the user manual. Briefly, 25µL of standards or samples were incubated together with freshly prepared working solution for 30 min at 60 °C in 96-well plates (Costar^®^ Corning Incorporated, NY, USA). Afterwards, the absorbance was measured at 570 nm with a plate-reader (Mithras LB 940 and MikroWin Version 4.41 software, both from Berthold Technologies, Bad Wildbad, Germany). The amount of EV protein/1 × 10^6^ CardAP cells was calculated.

### Surface marker expression analysis of cells and EVs by flow cytometry

The expression of surface markers on cells was investigated by staining with multicolour panels of human-specific fluorescence labelled antibodies according to the method previously described [76] and measured by flow cytometry. Three different antibody panels were used to stain immune cells. PHA stimulated and unstimulated PBMCs were stained with following human-specific antibodies: CD8-PE (1:200) (Miltenyi Biotec), CD14-APCCy7 (1:50), CD19-V450 (1:1000), CD3-PerCPCy5.5 (1:100) (BD Biosciences, San Jose, CA, USA), CD4-APC (1:100), CD56–PacificBlue™ (1:50) (BioLegend) and a viability marker in the V510 channel (1:100; LIVE/DEAD^®^ Fixable Aqua Dead Cell Stain Kit; Invitrogen/Thermo Fisher Scientific, Eugene, OR, USA). To evaluate the phenotype of CD14^+^ cells in PBMC cultures, collected PBMCs were stained with human-specific antibodies: CD86-PE (1:100), PD-L1-PerCPCy5.5 (1:50), HLA-DR-PECy7 (1:1000), CD206-APC (1:100), CD14-APCCy7 (1:50) and V510 viability marker. PBMCs and isolated T cells stimulated with anti-CD3 were stained with human-specific antibodies: CD19-V450 (1:1000), CD14-APCCy7 (1:50) (BD Bioscience), CD8 PECy7 (1:50), CD4-PerCPCy5.5 (1:75), CD56-PacificBlue™ (1:50) (BioLegend) and the V510 viability marker. Afterwards the cells were washed in FACS buffer, fixed in .5% PFA-supplemented FACS buffer and stored in the dark at 4 °C until measurement using the BD FACS-Canto II (BD Biosciences). Intracellular staining of Foxp3 was performed according the manual of Foxp3/Transcription Factor Staining Buffer Set (Invitrogen by Thermo Fisher Scientivic, Carlsbad, CA, USA). Firstly, cells were labelled on their surface as described above. Secondly, the cells were permeabilized and followed by an intracellular staining with anti-human Foxp3 AlexaFluor488 (1:400; BD Biosciences).

In contrast to cells, EVs were bound to 4 µm aldehyde/sulphate beads (Molecular Probes^®^, Life Technologies, Eugene, OR, USA) prior to staining. Therefore, 2 µg of EV-protein was incubated with 15 µL of beads in PBS for 1 h at room temperature. After a washing step, the beads were stained with the following human-specific antibodies: CD90-APC (1:50), CD44-PECy7 (1:100), CD73-APC (1:50), CD29-PE (1:200), CD63-PE (1:1000), CD81-FITC (1:1000), CD9-FITC (1:1000), CD106-PE (1:100), PD-L1-PerCPCy5.5 (1:50), CD54-APC (1:50), HLA-ABC-FITC (1:100) and HLA-DR-APC (1:50; all purchased from BioLegend, San Diego, CA, USA). Finally, samples were washed, fixed in .5% PFA-supplemented FACS buffer as described above and stored at 4 °C until measurement on a MACSQuant (Miltenyi Biotec).

All flow cytometry data were analysed with FlowJo 10.2. software (FlowJo™, LLC, RO, USA). In Additional file 1: Figure S5, the gating strategies for measuring T cell proliferation in PBMC and isolated T cell cultures as well as measuring surface proteins of EVs are shown.

### Mass spectrometry of CardAP-EVs

The protein composition of CardAP-EVs was analysed by liquid chromatography/electron spray ionization mass spectrometry (LC/ESI–MS) as described previously [77]. Therefore, CardAP-EVs from three different donors derived either under unstimulated or cytokine stimulation were transferred to amico filters (10kDA cut off, Merck, Darmstadt, Germany) followed by an overnight digestion with Trypsin (12 µg trypsin in 50 mM ammonium bicarbonate; Promega, Madison; WI USA) at 37 °C. Peptide samples were extracted with .1% trifluoroacetic acid (Fluka, St. Louis, USA) and measured by UPLC (Dionex Ultimate 3000, ThermoFisher, Waltham, MA, USA) ESI-QTOF mass spectrometer (Impact II, bruker daltonics, Billerica, MA, USA). The obtained mass spectra were analysed by searching the SwissProt database (human 553474 sequences, 198069095 residues, Cambridgeshire, UK) with MASCOT software (version number 2.2, Matrix Sience, Boston, MA, USA). The following parameters were set for analysis: (i) taxonomy: *Homo sapiens* (Human) (20175 sequences); (ii) proteolytic enzyme: trypsin; (iii) maximum of accepted missed cleavages: 1; (iv) mass value: monoisotopic; (v) peptide mass tolerance 10 ppm; (vi) fragment mass tolerance: .05 Da; and vii) variable modifications: oxidation. Identified proteins were considered for further analysis if scores corresponded to *p *<* .05* and if at least two donors showed at least one detected peptide. Networks of protein interactions were visualized with the help of String database (version 10.5 http://string-db.org) as connecting line. They were considered just for high confidence interaction (.77) of active interaction sources by experiments, databases, co-expression and co-occurrences.

### Immune cell isolation and purification

Peripheral blood mononuclear cells (PBMCs) were isolated from healthy blood donors by using a Biocoll gradient as described previously [76]. T cells (CD3^+^ cells) and monocytes (CD14^+^ cells) were enriched from the PBMCs by magnetic activated cell sorting (MACS). Here, PBMCs were incubated with human-specific CD3 or CD14 MicroBeads and isolated according to the manufacturer’s protocol with LS Columns (all from Miltenyi Biotec). The purity of separated cells ranged between 98.0 and 99.6% as determined by flow cytometry.

### Immune cell proliferation assay

PBMCs or isolated T cells were analysed in a carboxyfluorescein succinimidyl ester (CFSE)-based proliferation assay. Therefore, cells were labelled with 5 mM CFSE (Molecular Probes^®^, Life Technologies) in PBS for 3 min and washed twice in complete RPMI (Biochrom) medium containing 10% ultracentrifuged human serum, 1% penicillin/streptomycin and 1% l-glutamine (Gibco^®^ Life Technologies). In 96-well plates (costar^®^, Corning Incorporated) 3 × 10^5^ labelled PBMCs were seeded per well and applied either to 12.5 ng/mL of anti-CD3 antibody (OKT3; Janssen-Cilag, Neuss, Germany), .5 µg/mL phytohemagglutin (PHA, Sigma Aldrich, St. Louis, MO, USA) or left unstimulated. Unstimulated and anti-CD3 stimulated immune cells were treated with 12 µg/mL cytokine-stimulated or unstimulated EVs, while PHA stimulated PBMCs just received 6 µg/mL of EVs respectively. As controls, immune cells were treated with PBS in equal volume as EVs or left untreated. For the co-culture of isolated T cells and CD14^+^ cells, the CD14^+^ cells were 48 h prior to the assay treated with either CardAP-EV variant, PBS or left untreated. Afterwards, the cells were co-cultured with isolated T cells in a 1 to 5 ratio and treated with anti-CD3 as previously described. After three or 5 days, supernatants and immune cells were collected for further analysis.

## Detection of cytokines in immune cell cultures

Supernatants were analysed for IFNγ and active TGFβ using an enzyme-linked immunosorbent assay (ELISA; ELISA MAX™ Deluxe; BioLegend) according to the manufacturer’s protocol. Samples were measured at 450 nm and 570 nm on a plate reader (Mithras LB 940 and MikroWin Version 4.41 software, both from Berthold Technologies). The cytokines TNFα, IL-1β, IL-17a and IL-10, as well as the soluble receptor, IL-1RA, were evaluated using a multiplex bead-based assay (LEGENDplex™, BioLegend) according to the manufacturer’s protocol. Multiplex samples were measured by flow cytometry at a BD FACS-Canto II (BD Biosciences) and analysed with LEGENDplex™ version 7.1 (VigeneTech Inc, Carlisle, MA, USA).

### Determination of recipient cells for CardAP-EVs

EVs were labelled with 6 µL Vybrant^®^ DiD (Invitrogen™, Molecular Probes, Eugene, OR, USA) in 6 mL PBS for 10 min on ice prior to the final EV isolation step. A negative control (DiD^−^ control) without any EVs was processed in the same manner. After the centrifugation, both samples were reconstituted in 500 µL 0.1 µm filtered PBS and stored at − 80 °C. 5 × 10^5^ unlabelled PBMCs were treated with 12 µg DiD^+^EVs or equal volume of DiD^−^ control for 24 h at 37 °C. Afterwards, cells either were harvested for flow cytometric analysis as previously described or analysed by microscopy. Here, cells were washed with PBS, fixed with 4% PFA in PBS and labelled with human-specific CD14 APCCy7 antibody (BD Biosciences) and 4′,6-diamidino-2-phenylindole (DAPI; Molecular Probes^®^, Life Technologies). After two washing steps, cells were examined in 20× magnification with the help of an AxioObserver microscope running AxioVision software (both from Carl Zeiss Microscopy GmbH).

### Statistical analysis

All data are shown as median with interquartile range. Statistical differences were determined either for two groups using Mann–Whitney nonparametric t-tests or Wilcoxon matched-pairs signed rank test for paired samples. More than two groups were tested by a Friedman’s Test with Dunn´s Post Test for paired nonparametric samples. Results were considered significant with *p < .05, **p < .01, ***p < .001. Statistical analysis was performed using GraphPad Prism 6.0 software (GraphPad Software Inc, San Diego, CA, USA).

## Additional file

**Additional file 1.** Additional figures and table.

## Abbreviations

CardAPs: human cardiac-derived adherent proliferating cells
CD: cluster of differentiation
CPCs: cardiac progenitor cells
CVD: cardiovascular diseases
EVs: extracellular vesicles
IFNγ: interferon gamma
IL: interleukin
IL-1RA: interleukin 1 receptor antagonist
LC/ESI–MS: liquid chromatography/electron spray ionization mass spectrometry
MSCs: mesenchymal stromal cells
PBMCs: peripheral blood mononuclear cells
PBS: phosphate buffer saline
PHA: phytohemagglutin
TGFß: transforming growth factor beta
TNFα: tumour necrosis factor alpha

## Availability of data and materials

The datasets used and analysed during the current study are available from the corresponding author on reasonable request. In general, data generated or analysed during this study are included in this published article and its additional file.
