# Supplementary material for: Extracellular vesicles from regenerative human cardiac cells act as potent immune modulators by priming monocytes
Source: J Nanobiotechnology. 2019 May 27;17:72. doi: 10.1186/s12951-019-0504-0 (PMC6537224; doi:10.1186/s12951-019-0504-0)
Supplement: Supplementary file 1 — Additional file 1. Additional figures and table. [file 12951_2019_504_MOESM1_ESM.docx]

Additional File 1

**Extracellular Vesicles from Regenerative Human Cardiac Cells Act as Potent Immune Modulators by Priming Monocytes**

Christien M. Beez^1, 2^, Marion Haag^1, 3^, Oliver Klein^1, 6^, Sophie Van Linthout^1, 4, 5^, Michael Sittinger^1, 3^, Martina Seifert*^1, 2^ *****

**Correspondence:** Corresponding Author: martina.seifert@charite.de

**Additional Material & Methods**

## Culture of human cardiac-derived adherent proliferating cells

CardAP cells were grown at a density of 6000 cells/cm² in medium consisting of equal amounts of IMDM/DMEM/Ham´s F12 (IDH; all Biochrom, Berlin, Germany) and supplemented with 10% ultracentrifuged human serum, 1% penicillin/streptomycin (Gibco® Life Technologies, Grand Island, NY, USA), 20 ng/mL basic fibroblast growth factor and 10 ng/mL epithelial growth factor (both from Preprotech, Hamburg, Germany).

## Harvest of cells for flow cytometry and CardAP cell panel

Immune cells were harvested from the culture plates by application of Accutase (Gibco®, Life Technologies) for 15-30 minutes at 37°C and CardAP cells were detached by application of trypsin/EDTA (Gibco®, Life Technologies) for 3 minutes at 37°C. The process was stopped by adding double the amount of ucIDH medium. Then, the cells were directly transferred into 5 mL Polystyrene Round-Bottom Tubes (Falcon®, Corning Science México, Tamaulipas, Mexico) and washed once in FACS buffer consisting of PBS supplemented with 1% fetal calf serum (Clonetic®, Lonza, Wakersville, MD, USA). Then after, cells were stained according to the stated protocol. CardAP cells were stained for 30 minutes in the dark with human-specific antibodies: CD90-APC (1:50), CD44-PECy7 (1:100), CD73-APC (1:50), CD29-PE (1:200), CD63-PE (1:1000), CD81-FITC (1:1000), CD9-FITC (1:1000), CD106-PE (1:100), PD-L2-APC (1:50), PD-L1-PerCPCy5.5 (1:50), CD54-APC (1:50), HLA-ABC-FITC (1:100), HLA-DR-APC (1:50), CD86-PE (1:50), CD80-FITC (1:20), CD120c-PECy7 (1:50), CD119-PE (1:50; all purchased from BioLegend, San Diego, CA, USA) and CD121a-APC (1:50; R&D Systems, Minneapolis, MN, USA).

## *Additional Figures*


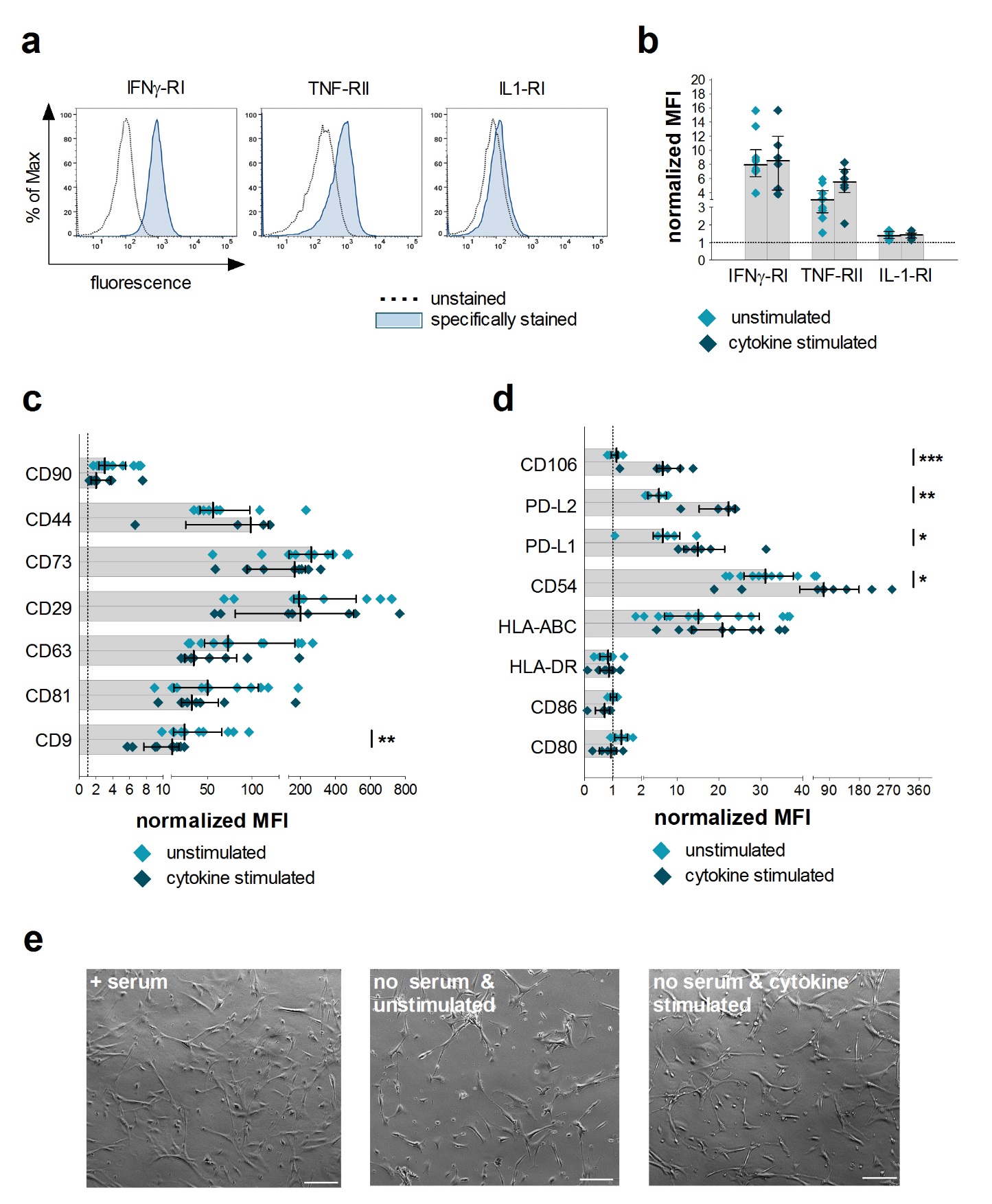


***Figure S1*:** CardAP cells respond towards pro-inflammatory cues during EV generation. For the generation of EVs, CardAP cells were cultured for 20 hours either in unstimulated or cytokine stimulated condition (10 ng/mL of IFNγ, TNFα and IL-1β). While the conditioned medium was collected for EV isolation, CardAP cells from both conditions were harvested, stained with human-specific fluorescence labelled antibodies and measured by flow cytometry. Detected geometrical mean fluorescence intensities (MFI) of surface markers were normalized to the unstained control by calculating the ratio between stained sample and unstained control. The normalized MFI for the unstained controls are indicated as dotted line in the graphs. **(a):** Representative histograms are shown for unstimulated CardAP cells in comparison to unstained control for the cytokine receptors IFNγ-RI, TNF-RII and IL-1-RI. **(b, c, d):** Normalized MFIs are shown for all three receptors **(b)**, for mesenchymal markers (CD90, CD73, CD29, CD44) and tetraspanins (CD9, CD81, CD63) **(c)** or for immunological relevant markers (CD54, CD80, CD86, CD106, HLA-ABC, HLA-DR, PD-L1 and PD-L2) **(d)** as median with interquartile range (n = 6 - 12; four to six different CardAP donors). **(e)**: Representative bright field images show the typical morphology of CardAP cells grown for 20 hours in medium with serum (left), without serum in unstimulated (middle) or cytokine-stimulated (right) condition. The scale bars represent 100 µm. Statistical significance was tested using Mann Whitney U-test; ***p < .001, **p < .01, *p < .05.

**
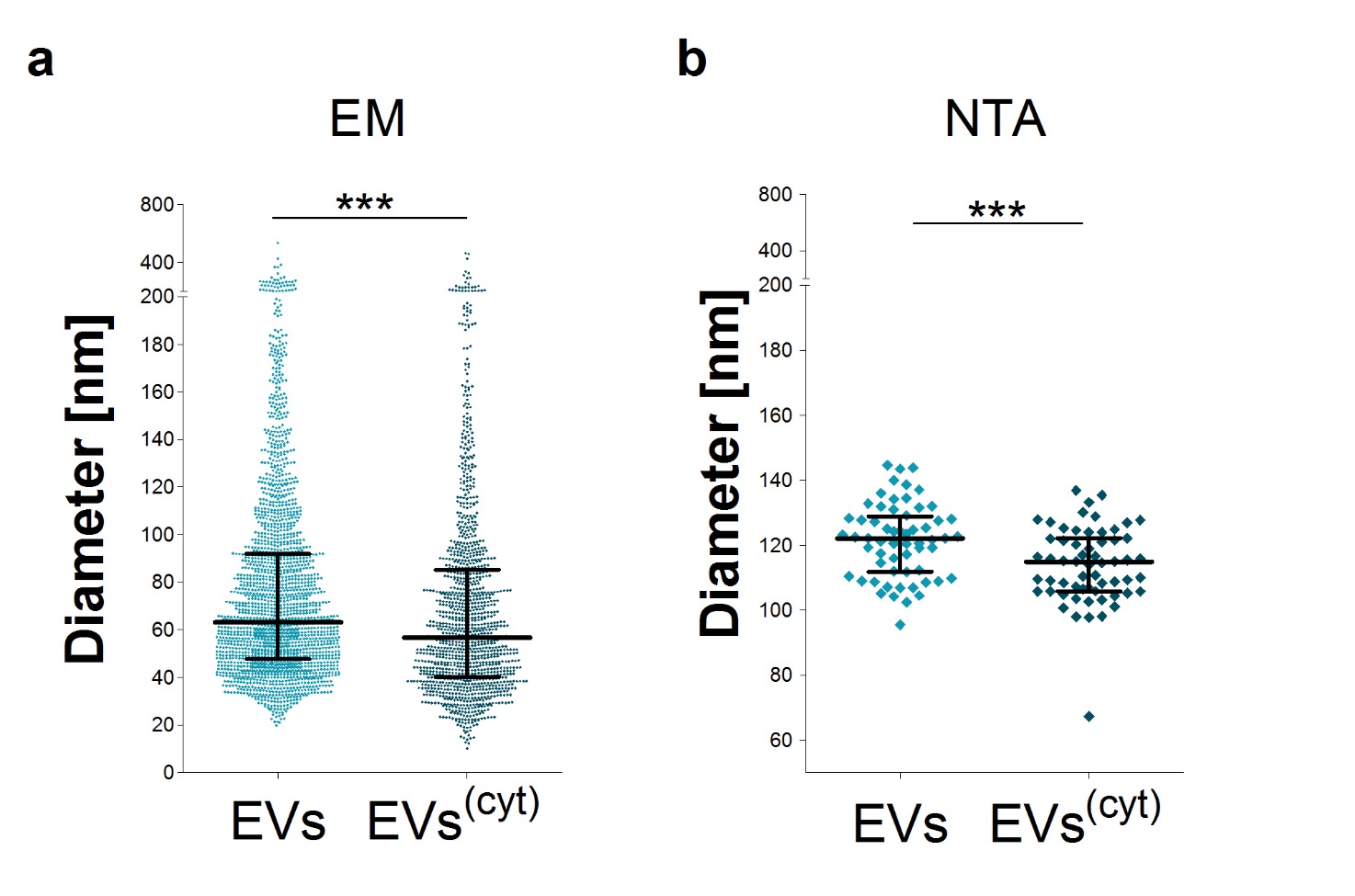
 *Figure S2*:** Cytokine-stimulated CardAP-EVs show a smaller diameter in comparison to unstimulated EVs as determined by transmission electron microscopy (TEM) and nanoparticle tracking analysis (NTA). EVs were generated by culturing CardAP cells either in unstimulated or cytokine stimulated conditions. After 20 hours, the conditioned medium was collected and used to isolate unstimulated EVs (EVs) and cytokine stimulated EVs (EVs^(cyt)^)**.**  **(a):** TEM pictures were evaluated for the diameter of both EV variants generated from three different CardAP donor. Per sample at least nine pictures were combined for analysis and obtained diameter are shown as median with interquartile range (n = 1200 - 2000; three different CardAP donors). **(b)**: By NTA measured diameters of both CardAP-EV variants are shown as median with interquartile range (n = 60, three different CardAP donors). Statistical significance was tested using Mann Whitney U-test; ***p < .001, **p < .01, *p < .05.

**
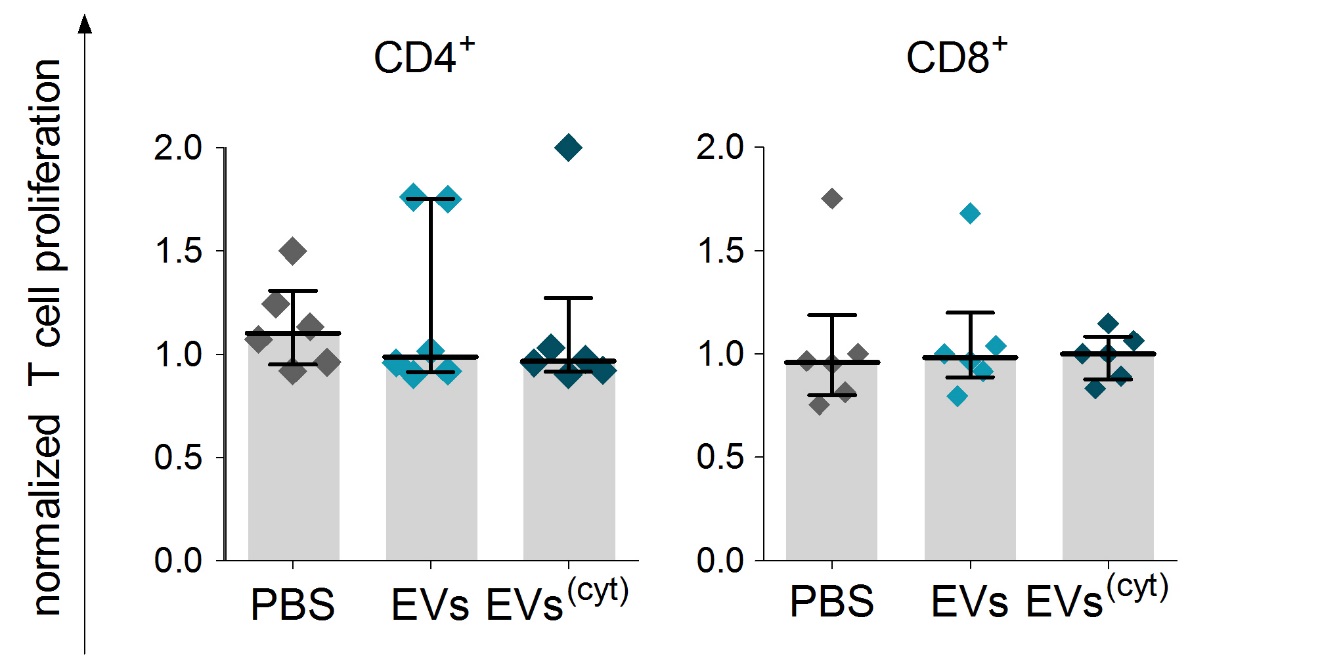
**

***Figure S3:*** Exposure of CardAP-EVs to PBMC cultures does not induce T cell proliferation. Isolated PBMCs were CFSE labelled, 3 x 10^5^ cells seeded in 96-well plates and exposed to 6 µg/mL unstimulated EVs (EVs), 6 µg/mL cytokine stimulated EVs (EVs^(cyt)^), PBS in equal volume of the EVs (PBS) or left untreated as a control. After 5 days, the immune cells were harvested, stained with human-specific fluorescence labelled antibodies and analysed by flow cytometry. Determined proliferation frequencies for CD4^+^ and CD8^+^ T cells were normalized for each treatment to the untreated control. The normalized proliferation of CD4^+^ T cells (left) and CD8^+^ T cells (right) is shown for treatments with PBS vs EVs or EVs^(cyt)^ median with interquartile range (n = 7; four different CardAP donors; four different PBMC donors).

*
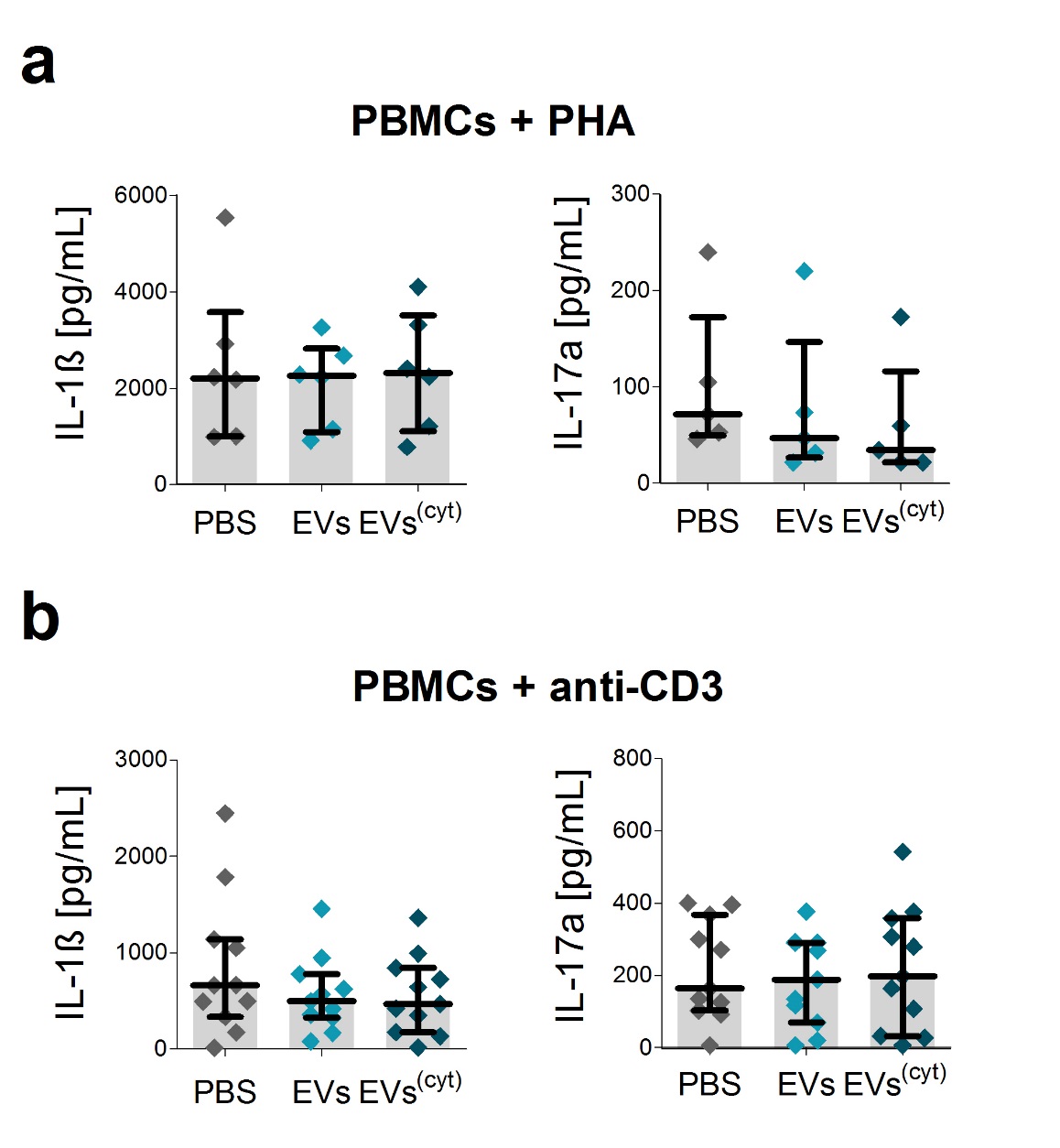
* ***Figure S4:*** CardAP-EVs did not affect the PHA and anti-CD3 induced pro-inflammatory cytokine release of IL-1ß and IL-17a in PBMC cultures. 3x10^5^ CFSE-labelled PBMCs were stimulated with PHA or anti-CD3 antibody and treated with either unstimulated (EVs) or cytokine stimulated (EVs^(cyt)^) EVs, PBS in equal volume of the EVs (PBS) or left untreated and analysed after 3 - 5 days. The cytokines of the supernatants were analysed by Multiplex (IL-17a, IL-1ß). **(a, b)** Concentrations for all tested cytokines are presented for PHA stimulated PBMC cultures **(a)** or anti-CD3 stimulated PBMC cultures **(b)** as median with interquartile range (PHA n = 6- 7, five different CardAP donors, five different PBMC donors) (anti-CD3 n = 8; four different CardAP donors, four different PBMC donors).


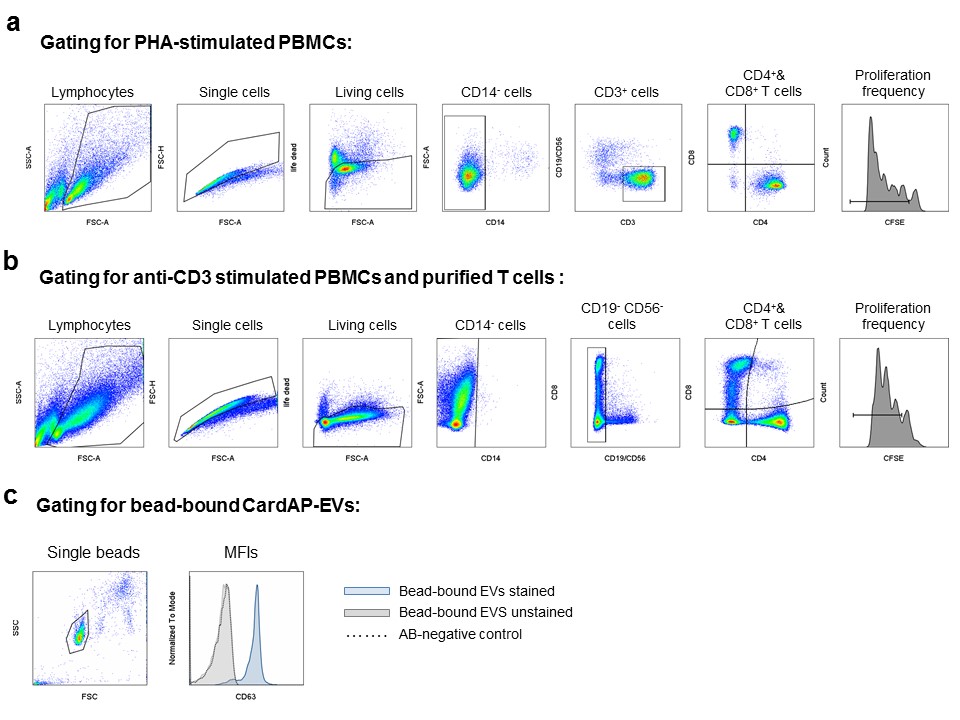
***Additional Material Figure S5*:** Gating strategy of immune cells and CardAP-EVs for flow cytometry. **(a, b):** Isolated PBMCs were CFSE labelled, 3 x 10^5^ cells were seeded in 96-well plates, stimulated with 0.5 µg/mL PHA or 12ng/mL anti-CD3 antibody under the additional treatment with CardAP-EVs, PBS or were left untreated. After 3 – 5 days, immune cells were harvested, stained with human-specific fluorescence labelled antibodies and analysed by flow cytometry. Following gating was used for PHA stimulated samples **(a):** events were gated (black lined/framed gate) on single living CD14^-^ CD3^+^ lymphocytes, which were then separated into CD4^+^ and CD8^+^ to evaluate the decrease of CFSE signal and to determine the frequency of proliferating T cells respectively. Anti-CD3 stimulated samples **(b)** were not able to stain sufficiently for CD3 surface molecules. Therefore, we gated on single living CD14^-^CD19^-^CD56^-^ lymphocytes and discriminated between CD4^+^ and CD8^+^ T cells. Accordingly, the decrease of CFSE signal was analysed and the frequency of proliferating T cells was determined respectively. **(c):** EVs were unspecifically bound to latex beads, washed and labelled with human-specific fluorescence labelled antibodies and analysed by flow cytometry. To determine the geometrical mean fluorescence intensity (MFI) it was necessary to gate on single beads and compare it with unstained control of bead-bound EVs as well as beads without EVs labelled for the specific antibody.

## *Additional Material Table*

***Table S1*:** The proteome of CardAP-EVs is enriched with proteins of the extracellular compartment. Peptides were derived from unstimulated EVs (unstimulated) and cytokine stimulated EVs (cytokine stimulated) by an overnight digestion with trypsin. Mass spectra obtained by liquid chromatography/electron spray ionization mass spectrometry (LC/ES MS) were evaluated by MASCOT software searching for protein matches in the SwissProt 51.9 database. The 186 proteins were analyzed with the help of String database to acquire information of their localisation as well as their involvement in biological processes. A blue cross (**x**) indicates the assignment to localization and/or processes when applicable.

|  | Assignment by String analysis: | | | | |  |
| --- | --- | --- | --- | --- | --- | --- |
| UniProt Identifier | Wound healing | Pos. regulation of biological processes | Angio-genesis | Regulation of immune system processes | Extra-cellular exosome | Complete name of the identified protein |
| 1433B |  | **x** |  | **x** | **x** | 14-3-3 protein beta |
| 1433E |  | **x** |  |  | **x** | 14-3-3 protein epsilon |
| 1433G |  | **x** |  |  | **x** | 14-3-3 protein gamma |
| 1433T |  | **x** |  |  | **x** | 14-3-3 protein theta |
| 1433Z | **x** | **x** |  |  | **x** | 14-3-3 protein zeta/delta |
| 2ABA |  | **x** | **x** |  |  | Serine/threonine-protein phosphatase 2A 55 kDa regulatory subunit B alpha isoform |
| 4F2 | **x** |  |  |  | **x** | 4F2 cell-surface antigen heavy chain |
| 5NTD |  | **x** |  |  | **x** | 5'-nucleotidase |
| AAAT |  |  |  |  | **x** | Neutral amino acid transporter B(0) |
| ABCE1 |  |  |  |  |  | ATP-binding cassette sub-family E member |
| ACLY |  | **x** |  |  | **x** | ATP-citrate synthase |
| ACTB | **x** | **x** |  | **x** | **x** | Actin; cytoplasmic 1 |
| ACTN1 | **x** |  |  |  | **x** | Alpha-actinin-1 |
| ACTZ |  |  |  |  |  | Alpha-centractin |
| ALDOA | **x** |  |  |  | **x** | Fructose-bisphosphate aldolase A |
| AMPN |  |  | **x** |  | **x** | Aminopeptidase N |
| ANXA1 | **x** |  |  | **x** | **x** | Annexin A1 |
| ANXA2 |  |  | **x** |  | **x** | Annexin A2 |
| ANXA4 |  |  |  |  | **x** | Annexin A4 |
| ANXA5 | **x** |  |  | **x** | **x** | Annexin A5 |
| ANXA6 |  |  |  |  | **x** | Annexin A6 |
| AP2A1 |  |  |  | **x** |  | AP-2 complex subunit alpha-1 |
| APOA1 | **x** | **x** |  | **x** | **x** | Apolipoprotein A-I |
| ARF1 |  | **x** |  | **x** | **x** | ADP-ribosylation factor 1 |
| ARP3 |  | **x** |  | **x** | **x** | Actin-related protein 3 |
| ARPC2 |  |  |  | **x** | **x** | Actin-related protein 2/3 complex subunit 2 |
| ARPC3 |  | **x** |  | **x** | **x** | Actin-related protein 2/3 complex subunit 3 |
| AT1A1 |  | **x** |  |  | **x** | Sodium/potassium-transporting ATPase subunit alpha-1 |
| AT2B1 | **x** |  |  |  | **x** | Plasma membrane calcium-transporting ATPase 1 |
| BGH3 |  |  | **x** |  | **x** | Transforming growth factor-beta-induced protein ig-h3 |
| CAB39 |  |  |  |  | **x** | Calcium-binding protein 39 |
| CALX |  |  |  |  | **x** | Calnexin |
| CAP1 | **x** | **x** |  |  | **x** | Adenylyl cyclase-associated protein 1 |
| CATB |  | **x** |  | **x** | **x** | Cathepsin B |
| CD276 |  |  |  |  | **x** | CD276 antigen |
| CDC42 | **x** | **x** | **x** | **x** | **x** | Cell division control protein 42 homolog |
| CDCP1 |  |  |  |  |  | CUB domain-containing protein 1 |
| CLH1 |  |  |  |  | **x** | Clathrin heavy chain 1 |
| CN37 |  |  |  |  | **x** | 2';3'-cyclic-nucleotide 3'-phosphodiesterase |
| CO3 | **x** | **x** |  | **x** | **x** | Complement C3 |
| CO6A1 |  |  |  |  | **x** | Collagen alpha-1(VI) chain |
| CO6A3 |  |  |  |  | **x** | Collagen alpha-3(VI) chain |
| COF1 | **x** |  |  | **x** | **x** | Cofilin-1 |
| CPNE1 |  | **x** |  |  | **x** | Copine-1 |
| CPNS1 |  | **x** |  |  | **x** | Calpain small subunit 1 |
| CTL2 |  | **x** |  |  | **x** | Choline transporter-like protein 2 |
| CTNA1 |  |  |  |  |  | Catenin alpha-1 |
| CTNB1 |  | **x** | **x** | **x** | **x** | Catenin beta-1 |
| DEST |  | **x** |  |  |  | Destrin |
| DPP4 |  | **x** |  | **x** | **x** | Dipeptidyl peptidase 4 |
| DSA2D |  |  |  |  |  | Putative dispanin subfamily A member 2d |
| DYHC1 |  |  |  |  | **x** | Cytoplasmic dynein 1 heavy chain 1 |
| DYSF | **x** |  |  |  | **x** | Dysferlin |
| EF1A1 |  |  |  |  |  | Elongation factor 1-alpha 1 |
| EF2 |  | **x** |  |  | **x** | Elongation factor 2 |
| EGLN | **x** | **x** | **x** |  |  | Endoglin |
| EHD2 | **x** | **x** |  |  | **x** | EH domain-containing protein 2 |
| ENOA |  | **x** |  |  | **x** | Alpha-enolase |
| EVA1B |  |  |  |  |  | Protein eva-1 homolog B |
| FAS |  | **x** |  |  | **x** | Fatty acid synthase |
| FLNA | **x** | **x** |  |  | **x** | Filamin-A |
| FSCN1 |  |  |  |  | **x** | Fascin |
| G3P |  |  |  |  | **x** | Glyceraldehyde-3-phosphate dehydrogenase |
| G6PD |  |  |  |  | **x** | Glucose-6-phosphate 1-dehydrogenase |
| GBG12 |  |  |  |  | **x** | Guanine nucleotide-binding protein G(I)/G(S)/G(O) |
| GDIB |  | **x** |  |  | **x** | Rab GDP dissociation inhibitor beta |
| GELS | **x** |  |  | **x** | **x** | Gelsolin |
| GNA11 | **x** |  |  |  | **x** | Guanine nucleotide-binding protein subunit alpha-11 |
| GNA13 | **x** | **x** | **x** |  | **x** | Guanine nucleotide-binding protein subunit alpha-13 |
| GNAI2 | **x** | **x** |  |  | **x** | Guanine nucleotide-binding protein G(i) subunit alpha-2 |
| GNAI3 | **x** |  |  |  | **x** | Guanine nucleotide-binding protein G(k) subunit alpha |
| GSTP1 |  | **x** |  |  | **x** | Glutathione S-transferase P |
| GTR1 |  |  |  |  | **x** | Solute carrier family 2; facilitated glucose transporter member 1 |
| H90B2 |  |  |  |  |  | Putative heat shock protein HSP 90-beta 2 |
| HS71A |  | **x** |  |  | **x** | Heat shock 70 kDa protein 1A |
| HSP7C |  |  |  |  |  | Heat shock cognate 71 kDa protein |
| IQGA1 |  | **x** |  |  | **x** | Ras GTPase-activating-like protein IQGAP1 |
| IST1 |  |  |  | **x** | **x** | IST1 homolog |
| ITA5 | **x** | **x** | **x** |  | **x** | Integrin alpha-5 |
| ITAV | **x** | **x** | **x** |  | **x** | Integrin alpha-V |
| ITB1 | **x** |  | **x** | **x** | **x** | Integrin beta-1 |
| ITM2B |  |  |  |  | **x** | Integral membrane protein 2B |
| K1C9 |  |  |  |  | **x** | Keratin; type I cytoskeletal 9 |
| K22E |  |  |  |  | **x** | Keratin; type II cytoskeletal 2 epidermal |
| K2C1 |  | **x** |  | **x** | **x** | Keratin; type II cytoskeletal 1 |
| K2C5 |  |  |  |  | **x** | Keratin; type II cytoskeletal 5 |
| KAP0 | **x** | **x** |  |  |  | cAMP-dependent protein kinase type I-alpha regulatory subunit |
| KPYM | **x** |  |  |  | **x** | Pyruvate kinase PKM |
| LAMA4 |  |  |  |  |  | Laminin subunit alpha-4 |
| LAMB1 |  | **x** |  |  | **x** | Laminin subunit beta-1 |
| LAMC1 |  | **x** |  |  | **x** | Laminin subunit gamma-1 |
| LAMP1 |  | **x** |  | **x** | **x** | Lysosome-associated membrane glycoprotein 1 |
| LAMP2 | **x** |  |  |  | **x** | Lysosome-associated membrane glycoprotein 2 |
| LDHA |  | **x** |  |  | **x** | L-lactate dehydrogenase A chain |
| LDHB |  |  |  |  | **x** | L-lactate dehydrogenase B chain |
| LEG1 |  |  |  | **x** | **x** | Galectin-1 |
| LG3BP |  |  |  |  | **x** | Galectin-3-binding protein |
| LOXL2 |  | **x** | **x** |  |  | Lysyl oxidase homolog 2 |
| LRC4C |  |  |  |  |  | Leucine-rich repeat-containing protein 4C |
| MAP1B |  | **x** |  |  |  | Microtubule-associated protein 1B |
| MMP14 |  |  | **x** |  |  | Matrix metalloproteinase-14 |
| MOES |  | **x** |  | **x** | **x** | Moesin |
| MVP |  |  |  |  | **x** | Major vault protein |
| MYH9 | **x** | **x** | **x** |  | **x** | Myosin-9 |
| MYL6 |  |  |  |  | **x** | Myosin light polypeptide 6 |
| MYL9 | **x** |  |  |  |  | Myosin regulatory light polypeptide 9 |
| MYO1B |  |  |  |  | **x** | Unconventional myosin-Ib |
| MYO1C |  | **x** |  | **x** | **x** | Unconventional myosin-Ic |
| NDKB |  |  |  |  | **x** | Nucleoside diphosphate kinase B |
| NIBL1 |  |  |  |  | **x** | Niban-like protein 1 |
| NID1 |  | **x** |  |  | **x** | Nidogen-1 |
| NID2 |  |  |  |  | **x** | Nidogen-2 |
| NRP1 |  | **x** | **x** |  |  | Neuropilin-1 |
| PA1B2 |  | **x** |  |  | **x** | Platelet-activating factor acetylhydrolase IB subunit beta |
| PAI1 |  |  |  |  |  | Plasminogen activator inhibitor 1 |
| PCBP1 |  |  |  |  | **x** | Poly(rC)-binding protein 1 |
| PCBP2 |  |  |  | **x** | **x** | Poly(rC)-binding protein 2 |
| PDC6I |  | **x** |  |  | **x** | Programmed cell death 6-interacting protein |
| PDIA1 |  |  |  |  | **x** | Protein disulfide-isomerase |
| PDIA3 |  |  |  |  | **x** | Protein disulfide-isomerase A3 |
| PGBM |  |  | **x** |  | **x** | Basement membrane-specific heparan sulfate proteoglycan core protein |
| PGK1 |  | **x** |  |  | **x** | Phosphoglycerate kinase 1 |
| PLAK |  | **x** |  |  | **x** | Junction plakoglobin |
| PLS1 | **x** | **x** |  | **x** | **x** | Phospholipid scramblase 1 |
| PLST |  |  |  |  |  | Plastin-3 |
| PLXB2 |  | **x** |  |  | **x** | Plexin-B2 |
| PPIA | **x** | **x** |  |  | **x** | Peptidyl-prolyl cis-trans isomerase A |
| PRDX5 |  | **x** |  |  | **x** | Peroxiredoxin-5; mitochondrial |
| PROF1 | **x** | **x** |  |  | **x** | Profilin-1 |
| PSME1 |  |  |  | **x** | **x** | Proteasome activator complex subunit 1 |
| PXDN |  |  |  |  | **x** | Peroxidasin homolog |
| RAB10 |  |  |  |  | **x** | Ras-related protein Rab-10 |
| RAB13 |  |  |  |  | **x** | Ras-related protein Rab-13 |
| RAB14 |  |  |  |  | **x** | Ras-related protein Rab-14 |
| RAB1A |  | **x** |  |  | **x** | Ras-related protein Rab-1A |
| RAB34 |  |  |  |  | **x** | Ras-related protein Rab-34 |
| RAB35 |  |  |  |  | **x** | Ras-related protein Rab-35 |
| RAB3B |  | **x** |  |  | **x** | Ras-related protein Rab-3B |
| RADI |  | **x** |  |  | **x** | Radixin |
| RALA |  | **x** |  |  | **x** | Ras-related protein Ral-A |
| RAP1A | **x** | **x** |  |  |  | Ras-related protein Rap-1A |
| RB22A |  |  |  |  | **x** | Ras-related protein Rab-22A |
| RFTN1 |  | **x** |  | **x** | **x** | Raftlin |
| RHOA | **x** | **x** |  |  | **x** | Transforming protein RhoA |
| RHOC | **x** | **x** |  |  | **x** | Rho-related GTP-binding protein RhoC |
| RRAS |  | **x** |  |  | **x** | Ras-related protein R-Ras |
| RRAS2 |  |  |  |  | **x** | Ras-related protein R-Ras2 |
| S10AB |  |  |  |  | **x** | Protein S100-A11 |
| S10AG |  |  |  |  | **x** | Protein S100-A16 |
| S12A2 |  |  |  |  | **x** | Solute carrier family 12 member 2 |
| S38A2 |  |  |  |  |  | Sodium-coupled neutral amino acid transporter 2 |
| SCRB2 |  |  |  |  | **x** | Lysosome membrane protein 2 |
| SDCB1 |  | **x** |  |  | **x** | Syntenin-1 |
| SEPR | **x** | **x** | **x** |  | **x** | Prolyl endopeptidase FAP |
| SEPT2 |  |  |  |  | **x** | Septin-2 |
| SERPH |  |  |  | **x** | **x** | Serpin H1 |
| STOM |  |  |  | **x** | **x** | Erythrocyte band 7 integral membrane protein |
| SYG |  |  |  |  | **x** | Glycine--tRNA ligase |
| SYWC |  |  | **x** |  | **x** | Tryptophan--tRNA ligase; cytoplasmic |
| TAGL2 |  |  |  |  | **x** | Transgelin-2 |
| TBA1A |  |  |  |  | **x** | Tubulin alpha-1A chain |
| TBB2A |  |  |  |  | **x** | Tubulin beta-2A chain |
| TBB5 |  |  |  |  | **x** | Tubulin beta chain |
| TBB6 |  |  |  |  |  | Tubulin beta-6 chain |
| TCPB |  |  |  |  | **x** | T-complex protein 1 subunit beta |
| TCPE |  |  |  |  | **x** | T-complex protein 1 subunit epsilon |
| TCPZ |  |  |  |  | **x** | T-complex protein 1 subunit zeta |
| TENA |  | **x** |  |  |  | Tenascin |
| TERA |  | **x** |  |  | **x** | Transitional endoplasmic reticulum ATPase |
| TGM2 |  | **x** |  |  | **x** | Protein-glutamine gamma-glutamyltransferase 2 |
| TLN1 | **x** |  |  |  | **x** | Talin-1 |
| TNAP3 |  | **x** |  | **x** | **x** | Tumor necrosis factor alpha-induced protein 3 |
| TNFA |  |  |  |  |  | Tumor necrosis factor |
| TPIS |  |  |  |  |  | Triosephosphate isomerase |
| TSN14 |  | **x** |  |  |  | Tetraspanin-14 |
| TSN3 |  |  |  |  | **x** | Tetraspanin-3 |
| TSP1 | **x** | **x** | **x** | **x** | **x** | Thrombospondin-1 |
| TTYH3 |  |  |  |  | **x** | Protein tweety homolog 3 |
| UBA1 |  |  |  |  | **x** | Ubiquitin-like modifier-activating enzyme 1 |
| VAT1 |  |  |  |  | **x** | Synaptic vesicle membrane protein VAT-1 homolog |
| VATB2 |  |  |  |  | **x** | V-type proton ATPase subunit B; brain isoform |
| VIME |  | **x** |  |  | **x** | Vimentin |
| VINC | **x** |  |  |  | **x** | Vinculin |
| VP37B |  | **x** |  |  | **x** | Vacuolar protein sorting-associated protein 37B |
| WDR1 | **x** | **x** |  |  | **x** | WD repeat-containing protein 1 |
| YES | **x** |  |  | **x** | **x** | Tyrosine-protein kinase Yes |
